# Metabolite profiling, antimalarial potentials of *Schleichera oleosa* using LC-MS and GC-MS: *in vitro*, molecular docking and molecular dynamics

**DOI:** 10.3389/fmolb.2025.1543939

**Published:** 2025-02-14

**Authors:** Peetha Vanaja, N. S. Hari Narayana Moorthy, Vivek Singh Rajpoot, Harshawardhan Rao, Rohit Kumar Goswami, Paranthaman Subash, Sulekha Khute, Kareti Srinivasa Rao

**Affiliations:** ^1^ Department of Pharmacy, Indira Gandhi National Tribal University, Amarkantak, India; ^2^ Department of Pharmacy, Sri Shanmugha College of Pharmacy, Sangagiri, India

**Keywords:** antimalarial, *in silico*, *in vitro*, phytoconstituents, *Schleichera oleosa*

## Abstract

**Purpose:**

To explore the phytochemical composition of *Schleichera oleosa* bark and evaluate its potential antimalarial activity through *in vitro* and *in silico* analyses.

**Methods:**

The bark of *S. oleosa* was subjected to Soxhlet extraction using petroleum ether, chloroform, and methanol. The quantitative analysis of the extracts was performed to determine total phenolic, flavonoid, and tannin contents. Advanced analytical techniques such as Gas Chromatography-Mass Spectrometry (GC-MS) and Liquid Chromatography-Mass Spectrometry (LC-MS) were employed to identify 175 phytoconstituents from the methanolic extract In-vitro antimalarial activity was assessed against Plasmodium falciparum using the candle jar method, measuring parasite growth inhibition. The inhibitory concentration (IC50) values were calculated and compared with standard antimalarial drugs, chloroquine and quinine. Furthermore, computational analyses, including molecular docking and molecular dynamics simulations, were conducted to evaluate the interactions of identified phytochemicals with key malarial targets (1CEQ and 4ZL4). The efficacy of these compounds was compared with standard drugs like artesunate and chloroquine. Additionally, ADMET (Absorption, Distribution, Metabolism, Excretion, and Toxicity) profiling and drug-likeness assessments were performed.

**Results:**

The methanolic extract of *S. oleosa* exhibited promising *in-vitro* antimalarial activity with an average IC50 value of 0.780 μg/mL, which, while higher than chloroquine (0.020 μg/mL) and quinine (0.268 μg/mL), still demonstrated significant efficacy. GC-MS and LC-MS analyses identified 175 phytoconstituents, among which two novel lead compounds, scillarenin and 4-[(Z)-(6-hydroxy-3-oxo-1-benzofuran-2(3H)-ylidene) methyl] phenyl beta-Dglucopyranoside, exhibited the highest docking scores and favorable ADMET profiles. Molecular docking and dynamics simulations confirmed strong binding affinities to malarial targets, surpassing some standard drugs in efficacy.

**Conclusion:**

This study reports, for the first time, the antimalarial potential of bioactive constituents derived from the bark of *S. oleosa*. The identified compounds, scillarenin and 4-[(Z)-(6-hydroxy-3-oxo-1-benzofuran-2(3H)-ylidene) methyl] phenyl beta-D-glucopyranoside, demonstrated promising antiplasmodial activity, validating traditional medicinal claims. The findings highlight the potential of *S. oleosa* as a source of novel antimalarial agents with fewer side effects compared to existing therapies. Further *in vivo* studies are warranted to confirm these results and support the development of new antimalarial drugs. This groundbreaking discovery contributes to the growing evidence supporting the role of medicinal plants in drug discovery.

## 1 Introduction

For thousands of years, plants have been used as medicines and have played a significant part in traditional medical practices across many countries. In response, scientists are investigating different phytoconstituents to discover possible treatments. Among these, plants have geared attention due to their rich diversity of bioactive substances. These include flavonoids, alkaloids, and terpenoids, which possess therapeutic properties. Flavonoids, known for their antioxidant effects, can help in reducing inflammation and boosting the immune response. Alkaloids, with their broad spectrum of pharmacological activities, may inhibit the growth of the malaria parasite. Terpenoids, often noted for their antimicrobial properties, offer potential in disrupting the lifecycle of the parasite. Herbal remedies have been used to address a variety of health issues since time immemorial, ranging from common colds to chronic diseases, and play a crucial role in complementary and alternative medicinal practices. Furthermore, about 50% of modern-day medications originate from plant sources., thus highlighting the ongoing importance of plants in the process of drug discovery and development (Raj and Jhariya, 2023).

*S. oleosa (Merr.) Oken.* Commonly known as Kusum, which contains 1,858 species. India has 72 plant species of the Sapindaceae family. It's a big, almost evergreen, deciduous tree with a somewhat short fluted trunk and can reach a height of 40 m *S. oleosa*, commonly known as Kusum, is recognized for its distinctive bark, which plays a crucial role in its identification and usage. The bark has been traditionally utilized in various cultures for its medicinal properties. It is typically rough and dark grey, often fissured with vertical cracks that give it a rugged appearance. This texture not only provides protection to the tree but also serves as a habitat for various microorganisms (Karthikeyan et al., 2023). This plant also has many reported pharmacological activities like anti-inflammatory activity, analgesic activity, anthelmintic activity, antibacterial activity, antioxidant activity, and reproductive activity (Goswami and Singh, 2017). According to phytochemical investigations, the bark of this plant contains scopoletin, beta-sitosterol, betulin, betulinic acid, lupeol and lupeol acetate (Bhatia et al., 2013). Additionally, another study has demonstrated that the outer bark of the *S. oleosa* plant contains tricadenic acid A and taraxerone (Ghosh et al., 2011). In addition to its approximately 10% tannin content, the bark is a source of significant anticancer agents, including betulin and betulinic acid. These compounds have geared an interest in its phytochemical research due to their potential therapeutic benefits.

Malaria, a potentially deadly disease caused by parasites from infected mosquitoes, affects nearly half of the world’s population, with approximately 3.3 billion people at risk of contracting the disease, according to the World Health Organization (Patel et al., 2024). It is predominantly affecting sub-Saharan Africa, also Asia, Latin America, and parts of Europe and the Middle East. Efforts to tackle malaria have made strides through various strategies, including mosquito nets treated with insecticides, indoor spraying, and effective antimalarial drugs. Research is ongoing to develop an effective malaria vaccine, which could play a crucial role in reducing the disease’s global burden. The pathogens that cause malaria have several enzymes and active pathways that serve as targets for antimalarial drugs. The treatment of malaria has been done using chloroquine and derivatives. Quinine, the first antimalarial medication, was extracted from the bark of the cinchona tree in the 1940s. Due to its affordability, effectiveness, and lower toxicity, chloroquine was the preferred treatment for malaria for many years (Unnissa et al., 2015). However, in modern malaria therapy, the use of chloroquine has been restricted due to the resistance developed in the malaria parasite. Instead of chloroquine, other noval derivatives like amodiaquine, mefloquine, and artemisinin were developed to fight against the plasmodium parasites to cure malaria.

The term anti-malarial activity describes a substance’s ability to prevent or cure malaria, a parasitic illness spread by the bites of infected mosquitoes. Antimalarial drugs work by targeting specific stages of the parasite’s life cycle inside the human body, particularly concentrating on the erythrocytic phase, which is responsible for the symptoms of malaria (Na-Bangchang and Karbwang, 2019). The WHO presently advises using artemisinin-based combination therapies (ACTs) as the primary treatment option for uncomplicated malaria caused by Plasmodium falciparum. However, the advent of ACTs has largely replaced the use of quinine as the first-line treatment due to their superior efficacy and reduced side effects. The parasite responsible for this disease, primarily Plasmodium falciparum, has shown an alarming capacity to develop resistance to existing antimalarial drugs such as chloroquine and ACTs. This resistance not only complicates treatment efforts, but also increases the risk of transmission and severe outcomes. Consequently, the scientific community is actively engaged in the discovery and development of novel therapeutic agents. Research has focused on identifying novel drug targets within the lifecycle of the parasite, leveraging advanced technologies, such as genomics and bioinformatics, to accelerate drug discovery. Additionally, alternative strategies, such as combination therapies and adjunctive treatments, are being explored to enhance the efficacy and prevent resistance. As the fight against malaria continues, innovation and dedication in the field of antimalarial research remain crucial in the quest to eradicate this debilitating disease.

*In-silico* methods are computational techniques and simulations used to model, analyze, and predict biological and chemical processes. This approach leverages the power of computer models to predict the behavior of molecules, interactions, and reactions, often before any physical experiments are conducted. In the process of drug discovery and development of new drugs, *in silico* techniques are frequently employed to identify promising therapeutic candidates, forecast their effectiveness, and evaluate their safety profiles. The primary advantage of *in silico* methods is their ability to rapidly analyze large datasets and generate hypotheses that can be tested *in vitro* and *in vivo*, thereby saving time and resources. As computational power continues to grow, *in silico* methodologies are becoming increasingly integral to modern scientific research and development.

*In vitro* studies allow researchers to examine the efficacy of plant extracts or compounds against the malaria parasite in a controlled laboratory environment. This method is crucial for identifying active ingredients that can potentially inhibit the growth and survival of the parasite. Additionally, *in silico* methods, which involve computer-based simulations and modeling, provide an efficient way to predict the interaction between plant-derived compounds and the molecular targets of the malaria pathogen. These approaches not only accelerate the identification of promising antimalarial agents but also help in understanding the underlying mechanisms of action (Boniface et al., 2015). Malarial proteins were selected based on previous reports like 1CEQ (*P. falciparum* lactate dehydrogenase with NADH binding site) (Kalani et al., 2013), 4ZL4 (plasmepsin V from *P. vivax* bound to a transition state mimetic) (Onguéné et al., 2014). The study focused on a comprehensive analysis of chemical constituents in the bark of *S. oleosa*, specifically phenols, flavonoids, and tannins, utilizing advanced techniques like GC-MS, LC-MS and molecular dynamics. GC-MS and LC-MS are critical tools for identifying and quantifying compounds in complex mixtures and provide detailed molecular information that is essential for understanding the pharmacokinetics and metabolism of potential drug candidates. GC-MS is particularly useful for volatile compounds, whereas LC-MS excels in the analysis of larger, non-volatile molecules, offering high sensitivity and specificity (Kumar et al., 2024). Molecular dynamics simulations complement these techniques by providing insights into the structural and functional dynamics of biological molecules at the atomic level (Huggins et al., 2019). This computational approach helps to predict how potential drugs interact with their targets, allowing for the optimization of binding affinities and the identification of promising therapeutic candidates. Together, these technologies form a powerful toolkit for drug discovery, enabling researchers to efficiently screen, identify, and refine new drug candidates with greater precision and speed (Duarte et al., 2019).

Malaria remains a global health challenge that requires the discovery of novel therapeutic agents to combat drug-resistant Plasmodium strains (Okombo and Fidock, 2024). In the present study, we have investigated the potential of secondary metabolites from the bark of *Schleichera* oleosa as anti-malarial agents. We hypothesized that these compounds exhibit significant therapeutic properties through their interaction with specific malaria-related molecular targets. To test this hypothesis, a combination of *in vitro* and *in silico* approaches were used. Molecular docking was performed to evaluate the binding efficiency and intermolecular interactions, followed by ADMET prediction to assess the pharmacokinetic properties of the compounds. Additionally, molecular dynamics simulations were conducted to observe the stability and dynamics of these interactions over time. By addressing these aspects, this study aimed to elucidate the therapeutic potential of *S. oleosa* constituents and contribute to the development of novel antimalarial drugs.

## 2 Results and discussion

### 2.1 Yield of extraction

The extract was placed in a container that had already been weighed, and its weight was documented. To find the net weight of the extract, the weight of the empty container was subtracted from this measurement. The percentage yield was then determined by dividing the extract’s weight by the initial sample’s weight. For *S. oleosa*, the extractive values in petroleum ether, chloroform, and methanol were found to be 0.1618 g, 0.115 g, and 4.1385 g, respectively. Because methanol is highly polar, it was able to extract more components, resulting in higher extractive values with methanol compared to the other solvents.

### 2.2 Quantitative analysis

**FIGURE 1:**
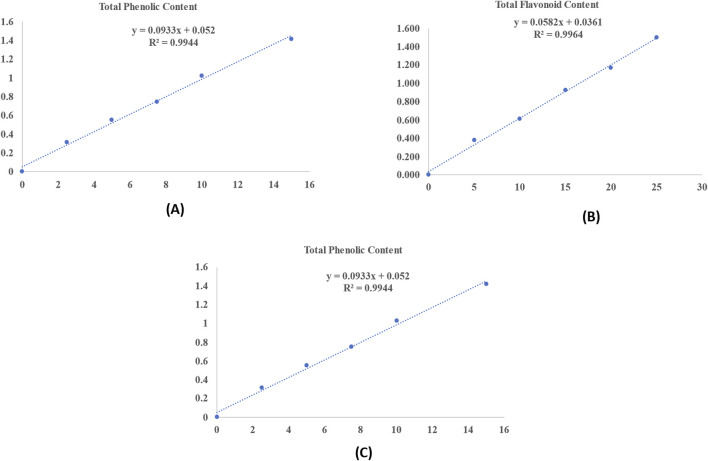
Standard curve of different metabolites: **(A)** Total phenolic content; **(B)** Total flavonoid content; and **(C)** Total tannin content.

To evaluate these components, quantitative analyses were performed on three distinct extracts to determine their total phenolic content (TPC), total flavonoid content (TFC), and total tannin content (TTC). The findings for the TPC, TFC, and TTC of *S. oleosa* bark are detailed in Table 1, with standard graphs displayed in Figure 1. The methanolic extract of *S. oleosa* bark contained higher levels of tannin, flavonoid, and phenol compared to the other two other extracts like petroleum and chloroform (Kyaw et al., 2019; Thind et al., 2011). In this study, we report for the first time a quantitative analysis of the total flavonoid and tannin content of the methanolic extract of the bark of *S. oleosa*. Reddy et al. (2007) highlighted the anti-malarial properties of tannins extracted from *Punica granatum*, which is commonly known as pomegranate. Tannins are polyphenolic compounds known for their astringent properties and have been recognized in various traditional medicines for their therapeutic benefits (Pizzi, 2021). These compounds may inhibit the growth of Plasmodium species, the parasites responsible for malaria, by interfering with their lifecycle. This discovery opens new avenues for further exploration of how natural compounds can be harnessed to combat malaria, a disease that continues to affect millions worldwide.

**TABLE 1:** Quantitative tests with quantity of phytoconstituent present in each solvent.

S. No	Quantitative test	% Of PE	% Of CE	% Of ME
1	Total Phenolic Content	__	10.26%	64.09%
2	Total Flavonoid Content	24.68%	21.22%	60.37%
3	Total Tanin Content	__	6.03%	67.06%

### 2.3 GC-MS and LC-MS analysis

**FIGURE 2:**
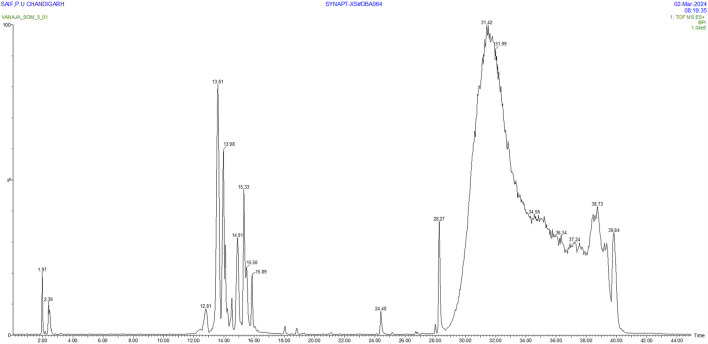
GC-MS Chromatogram of methanolic extract of *S. oleosa* bark: 151 phytoconstituents.

**FIGURE 3:**
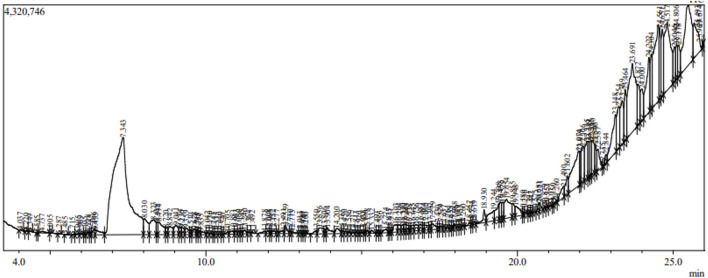
LC-MS Chromatogram of methanolic extract of *S. oleosa* bark: 24 phytoconstituents.

The analysis of *S. oleosa* bark’s methanolic extract using GC-MS and LC-MS provides a comprehensive understanding of its phytoconstituents. The GC-MS analysis identified 151 compounds within the extract, demonstrating the technique’s capability to separate and identify a wide range of volatile and semi-volatile compounds. Similarly, through the LC-MS analysis identified 24 compounds, focusing on those with sharp peaks, which often represent more stable, non-volatile compounds that are better suited for liquid chromatography. Figures 2, 3 likely illustrate the chromatograms obtained from these analyses, showing peaks that correspond to different phytoconstituents. Supplementary Table S1 appears to provide detailed information of identified phytoconstituents from GCMS and LCMS. Both methodologies utilize mass spectroscopy to identify compounds through their fragmentation patterns. This process involves comparing the observed patterns with existing spectral databases to accurately deduce the structure and identity of the compounds present. The exploration of phytoconstituents from plants such as *Hibiscus cannabinus*, *Corchorus capsularis*, and *Tetrapleura tetraptera* has shown promising results against malaria through both *in silico* and *in vitro* methods. Studies by Brahma et al. (2024) and Hamidu et al. (2023) have highlighted the potential of these plants as sources of antimalarial compounds. These investigations used computational models to predict interactions with malarial targets, followed by laboratory experiments to validate these findings.

This dual approach of using GC-MS and LC-MS allows for a more robust and detailed characterization of the phytochemical profile of *S. oleosa*, highlighting its potential pharmacological and therapeutic applications. The compounds scillarenin and 4-[(Z)-(6-hydroxy-3-oxo-1-benzofuran-2(3H)-ylidene) methyl] phenyl beta-D-glucopyranoside have shown promising potential in combating malaria, as evidenced by their high binding affinity to malaria targets in molecular docking studies. Following this discovery, these compounds underwent comprehensive analyses to evaluate their drug-likeness, which involved assessing their physicochemical properties and compliance with criteria such as Lipinski’s Rule of Five, indicating their potential as orally active drugs. Additionally, ADMET studies were also conducted to predict the pharmacokinetic profiles and safety of these compounds to ensure that they possessed favorable characteristics for therapeutic use. Furthermore, their stability was examined under molecular dynamics conditions to verify their structural integrity and performance under physiological conditions. These analyses provide a robust foundation for developing an effective anti-malarial agents from *S. oleosa*.

### 2.4 *In vitro* antimalarial activity

The study aimed to assess the antimalarial properties of a methanolic plant extract against *Plasmodium falciparum* using the Rieckmann micro-assay method (Rieckmann et al., 1968). After a 38-h incubation period, researchers counted the average number of rings, trophozoites, and schizonts per 100 parasites from duplicate wells. The effectiveness of the plant extract was measured by comparing the percentage of maturation inhibition to a control group. Remarkably, the extract demonstrated a minimum inhibitory concentration (MIC) of 0.78 μg/mL, with an IC50 value matching this MIC, indicating strong inhibitory potential against the malaria-causing parasite. This finding suggests that the plant extract could be a promising candidate for developing new antimalarial treatments, offering a potential alternative to existing therapies. *In vitro* systems, which are invaluable in scientific research, have notable limitations that must be considered when interpreting their results. The primary drawback is the inability to fully mimic intricate interactions within living organisms. These systems typically focus on 1 cell type or a limited combination of cell types, which restricts their ability to replicate complex cellular interactions and physiological processes *in vivo*. This limitation can affect their utility in comprehensive pharmacokinetic or toxicokinetic studies, as they cannot accurately simulate the distribution, metabolism, or excretion of substances within an entire organism. Despite these constraints, *in vitro* systems remain valuable for examining specific cellular responses, such as changes in morphology, particle uptake, cell signalling, gene expression, and protein production, which often correlate with *in vivo* outcomes (Stone et al., 2009).

In the present study, the average IC50 value of the methanolic extract (0.780 μg/mL), which was found to be higher than that of the standard drugs, chloroquine (0.020 μg/mL) and quinine (0.268 μg/mL), however, confirming its antimalarial efficacy. This diminished efficacy could be attributed to the concentration of phytoconstituent(s) which is/are responsible for antimalarial property in the crude extract may be very low or the net antimalarial property of the extract may not be due to a single phytoconstituent, rather it may be due to a mixture of phytoconstituents present in low concentration in the crude extract or the bioavailability of active compounds in the extract may very be low. The potential antimalarial properties of bark extract may be linked to the presence of phenols, flavonoids, and tannins (Goswami and Singh, 2017). These phytoconstituents are known for their antioxidant and antimicrobial activities, which could contribute to combating malaria (Khandekar et al., 2015). However, due to the complex nature of malaria treatment and the need for precise action against the parasite, the extract may not achieve the same level of effectiveness as established pharmaceuticals. Further research could explore optimizing extraction methods or combining plant-based compounds with standard treatments to enhance their efficacy.

### 2.5 Molecular docking

AutoDock Vina is a widely-used molecular docking software that facilitates the study of interactions between proteins and various compounds, aiding in drug discovery and design. In this context, the proteins 1CEQ and 4ZL4 were sourced from the Protein Data Bank to serve as targets for docking studies. The docking process involves evaluating 175 phytoconstituents, with their scores derived from GCMS and LCMS analyses illustrated in Supplementary Table S1. These scores reflect the stability and affinity of the compounds at the protein binding sites. Typically, compounds with lower docking scores are considered more stable and potentially more effective due to their stronger binding affinity. Conversely, compounds that exhibit higher docking scores than the standards are highlighted in bold, indicating a potential deviation in binding efficiency. In molecular docking studies, identifying the most favorable docking pose is crucial for understanding the interactions between a ligand and its target protein. The lowest binding energy conformation is often considered the most stable and thus most likely to occur in nature. This energy was calculated as the sum of the total intermolecular energy, total internal energy, and torsional free energy, with the energy of the unbound system subtracted, providing an accurate estimate of the binding affinity. In this process, tools such as AutoDock are used to generate multiple conformations of the ligand, typically selecting the top ones based on their energy values (Hasan et al., 2015). The top 16 ligand conformations were generated based on the binding energy value using AutoDock. The conformation with the lowest binding energy is typically considered to be the most favorable because it suggests a more stable interaction (Chaniad et al., 2016). This conformation is often found in the most populated cluster, indicating that it is not only energetically favorable but also statistically significant. Analyzing this conformation allows researchers to gain insights into key interactions, such as hydrogen bonds, hydrophobic interactions, and electrostatic forces, which contribute to binding affinity and specificity. Structural biology has seen significant advancements, particularly through the integration of computational methods, such as structure-based virtual high-throughput screening, which plays a critical role in drug discovery and development. This approach allows researchers to efficiently identify potential drug candidates by simulating their interactions with target proteins. However, despite these advances, current docking methodologies have several limitations. For instance, accurately modeling the flexibility of ligands and proteins remains challenging, which can affect the precision of binding predictions. Additionally, entropic effects, solvation/desolvation processes, and the presence of water molecules and ions further complicate the simulations. The existence of tautomers, allosteric effects, and the molecular context of the binding sites also add layers of complexity. Achieving binding and pharmacokinetic effects is crucial for developing effective therapeutics. Addressing these challenges requires the continual refinement of computational models and methodologies to improve drug discovery accuracy and reliability.

#### 2.5.1 Phytoconstituents with top docking score for 1CEQ protein target

The methanolic extract of *S. oleosa* bark has demonstrated promising potential in terms of binding affinity when compared to the standard reference compound, chloroquine, which has a binding affinity of −8. This suggests that ten compounds might have a stronger interaction with the target site, indicating potential therapeutic benefits such as Isorhamnetin 3-glucoside (−8.8), (+)-epicatechin-3-O-gallate (−8.8), (−)-Epicatechin-3-O-gallate (−8.7), Scillarenin (−8.6), (3beta, 17xi)-Stigmast-5-en-3-yl D-glucopyranoside (−8.6), (1Z, 2E)-1-({[(2R,3S,4S,5R, 6S)-6-{[5,7-Dihydroxy-2-(4-oxo-2,5-cyclohexadien-1-ylidene)-2H-chromen-3-yl] oxy}-3,4,5-trihydroxytetrahydro-2H-pyran-2-yl] methyl} oxonio)-3-(4-hydroxyphenyl)-2-propen-1-olate (−8.6), 5-(beta-D-Glucopyranosyloxy)-2-(4-hydroxyphenyl)-7-chromeniumolate (8.2), (1S)-1,5-Anhydro-1-(5,7-dihydroxy-4-oxo-2-phenyl-4H-chromen-8-yl)-D-glucitol (−8.1), 4-[(Z)-(6-Hydroxy-3-oxo-1-benzofuran-2(3H)-ylidene) methyl] phenyl beta-D-glucopyranoside (−8.1) and dUDP (−8.1).

#### 2.5.2 Phytoconstituents with top docking score for 4ZL4 protein target

The study on the methanolic extract of *S. oleosa* bark reveals that several phytoconstituents demonstrate a higher binding affinity than chloroquine, which has a binding affinity of −8.1. This suggests that fifteen compounds might have a stronger interaction with the target site, indicating potential therapeutic benefits such as (1Z, 2E)-1-({[(2R,3S,4S,5R, 6S)-6-{[5,7-Dihydroxy-2-(4-oxo-2,5-cyclohexadien-1-ylidene)-2H-chromen-3-yl] oxy}-3,4,5-trihydroxytetrahydro-2H-pyran-2-yl] methyl} oxonio)-3-(4-hydroxyphenyl)-2-propen-1-olate (−9.9), 5-(beta-D-Glucopyranosyloxy)-2-(4-hydroxyphenyl)-7-chromeniumolate (−9.2), Isorhamnetin 3-glucoside (−9), (+)-epicatechin-3-O-gallate (−9), 4-[(Z)-(6-Hydroxy-3-oxo-1-benzofuran-2(3H)-ylidene) methyl] phenyl beta-D-glucopyranoside (−9), Glutathione (−9), (3beta, 17xi)-Stigmast-5-en-3-yl D-glucopyranoside (−8.6), Paromomycin (−8.6), 2-Oxazolamine, 4,5-dihydro-5- (phenoxy methyl)-N-[(phenylamino)carbonyl]- (−8.4), Scillarenin (−8.3), (1S)-1,5-Anhydro-1-(5,7-dihydroxy-4-oxo-2-phenyl-4H-chromen-8-yl)-D-glucitol (−8.3), aloesin (−8.2), tetrapentacontane, 1,54-dibromo- (−8.2), magnesium 3-[8-(1-hydroxy-3-methoxy-3-oxopropyl)-2,7,13,17-tetramethyl-3,18-divinyl-21H-porphin-24-id-12-yl] propanoate (−8.2) and (−)-epicatechin-3-O-gallate (−8.2). These findings suggest that these phytoconstituents could potentially offer more effective binding interactions compared to chloroquine, indicating their potential as candidates for further investigation in the development of therapeutic agents.

### 2.6 Drug likeness and ADMET studies

Drug likeness and ADMET studies are crucial in the drug development process as they assess the Absorption, Distribution, Metabolism, Excretion, and Toxicity of potential compounds illustrated in Table 2. In this study, based on the higher docking score of ligands than the standard chloroquine, sixteen ligands with high-binding affinity were evaluated using the ADMET study and drug likeness characters. The evaluation of ADMET properties plays a crucial role in drug development, influencing decisions at every stage, from hit selection to preclinical development. Despite significant advances in the assessment of ADMET properties, several limitations hinder this process. The major challenge is the discrepancy between the *in vitro* and *in vivo* results. Although *in vitro* models provide valuable insights, they often fail to fully replicate the complex biological environments of living organisms. Algorithm limitations also pose a challenge, as the computational models used to predict ADMET properties may not always accurately reflect real-life scenarios. Furthermore, the physiological relevance of *in vitro* models is sometimes limited because they do not account for factors such as human-specific metabolic pathways. Lastly, variability in absorption can complicate predictions because individual differences in physiology can lead to significant variability in drug adsorption (Chandrasekaran et al., 2018). These factors are vital in determining the most suitable route of administration for drug formulations. ADMET properties help to predict how a drug behaves in the body, thereby influencing its efficacy and safety. Absorption determines how well a drug is taken up into the bloodstream; distribution assesses how it spreads through tissues; metabolism examines how the body processes it, often into active or inactive forms; excretion focuses on how the drug and its metabolites are eliminated; and toxicity evaluates the potential for adverse effects (Chandrasekaran et al., 2018). Among them sixteen phytoconstituents, nine phytoconstituents were common for both the targets. Only two compounds identified as scillarenin and 4-[(Z)-(6-Hydroxy-3-oxo-1-benzofuran-2(3H)-ylidene) methyl] phenyl beta-D-glucopyranoside, exhibited the highest docking scores for the selected targets. This suggests that these two phytoconstituents have significant potential for further development as therapeutic agents, due to their strong binding affinity, good ADMET properties and favorable drug-likeness properties. As a result, these two phytoconstituents have been selected for further investigation due to their promising ADMET profiles and drug-likeness properties from the methanolic extract of *S. oleosa* bark.

**TABLE 2:** Drug likeness and ADMET property of the selected top ligands from methanolic bark extract of *S. oleosa* and reference standard drugs.

Ligand No	Lead hit for target (s)	Drug likeness						ADMET property			
		MR 40–130	MW <500	HBD <5	HBA <10	LogP <5	RO5	BBB	PPB (%)	WS log (mol/L)	Carcinogenicity
4	4ZL4	87.7083	311	2	6	2.641	100%	96.70%	82.00%	−4.038	36.10%
58	4ZL4	136.3873	615	18	19	−8.861	0%	10.09%	0.00%	−0.515	23.80%
100	1CEQ, 4ZL4	105.7045	384	2	4	3.964	**100%**	89.90%	81.59%	−4.953	35.50%
134	4ZL4	267.672	914	0	0	22.061	20%	93.60%	95.30%	−8.535	51.58%
152	1CEQ	72.7088	388	5	13	−3.045	80%	18.30%	62.109	0.632	24.10%
153	1CEQ, 4ZL4	145.9851	580	7	12	2.101	20%	4.90%	98.10%	−3.568	52.00%
157	4ZL4	71.2743	307	6	9	−2.206	80%	15.50%	9.50%	−0.568	46.43%
159	4ZL4	93.9034	394	5	9	−0.346	100%	44.20%	32.50%	−2.295	82.25%
163	1CEQ, 4ZL4	101.8692	416	6	9	0.228	80%	11.60%	77.40%	−3.409	65.78%
164	1CEQ, 4ZL4	0	416	5	9	0	80%	56.80%	55.10%	−2.396	57.29%
165	1CEQ, 4ZL4	100.9284	416	5	9	0.1	**100%**	69.49%	65.80%	−3.152	79.43%
167	4ZL4	166.3394	604	3	8	2.983	60%	40.10%	98.50%	−5.787	58.20%
168	1CEQ, 4ZL4	111.161	478	7	12	−0.427	60%	39.80%	47.30%	−2.309	44.18%
169	1CEQ, 4ZL4	107.256	442	7	10	2.527	80%	6.20%	90.00%	−2.922	47.80%
170	1CEQ, 4ZL4	107.256	442	7	10	2.527	80%	6.20%	90.00%	−2.922	47.80%
171	1CEQ, 4ZL4	160.8503	576	4	6	5.849	40%	50.90%	98.00%	−6.711	67.63%
Chloroquine		96.8596	319.5	1	3	4.81	**100%**	95.80%	85.10%	−4.426	59.12%
Artemisinin		77.2728	300	3	6	2.608	**100%**	22.60%	100.00%	−3.63	76.90%

MR, Molar refractivity; MW, molecular weight; HBD, Hydrogen-bond donor; HBA, Hydrogen-bond acceptor; LogP, Partition coefficient; R05, Lipinski’s rule of five; PPB, Plasma Protein Binding; WS, Water solubility.

Bold values indicate 100% drug-likeness and common for both the selected targets.

### 2.7 Intermolecular interactions

**FIGURE 4:**
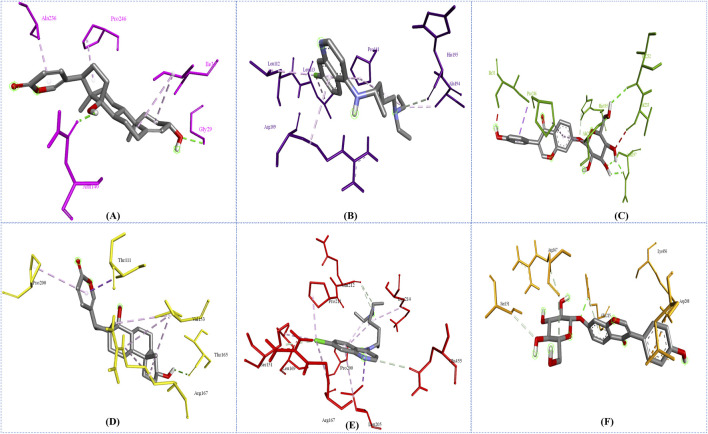
Binding site prediction through the Discovery studio: 1CEQ–Ligand 100 **(A)**, 1CEQ–standard chloroquine **(B)**, 1CEQ–Ligand 165 **(C)**, 4ZL4 – Ligand 100 **(D)**, 4ZL4 – standard chloroquine **(E)** and 4ZL4 – Ligand 165 **(F)**.

**FIGURE 5:**
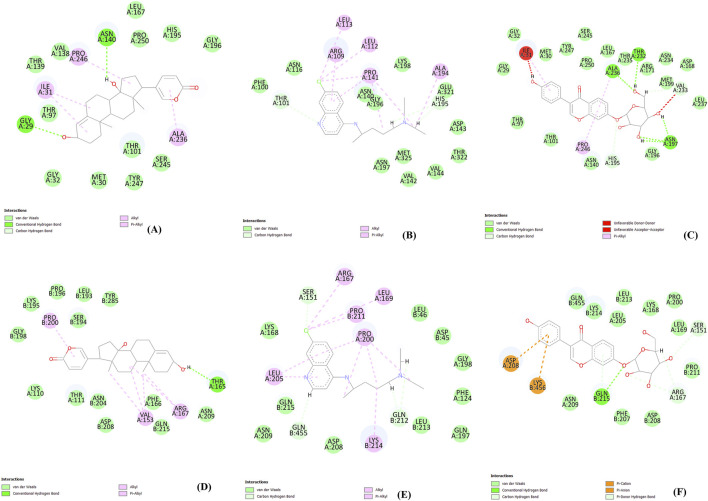
2D interactions: 1CEQ–Ligand 100 **(A)**, 1CEQ–standard chloroquine **(B)**, 1CEQ–Ligand 165 **(C)**, 4ZL4 – Ligand 100 **(D)**, 4ZL4 – standard chloroquine **(E)** and 4ZL4 – Ligand 165 **(F)**.

**FIGURE 6:**
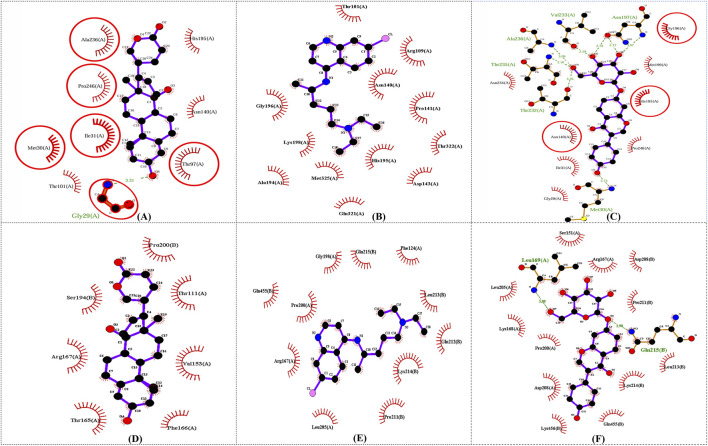
Intermolecular interactions through the LigPlot analysis: 1CEQ–Ligand 100 **(A)**, 1CEQ–standard chloroquine **(B)**, 1CEQ–Ligand 165 **(C)**, 4ZL4 – Ligand 100 **(D)**, 4ZL4 – standard chloroquine **(E)** and 4ZL4 – Ligand 165 **(F)**.

In this study, to examine intermolecular interactions, Discovery Studio and LigPlot were employed to analyze ligand 100, standard chloroquine, and ligand 165. These tools are instrumental in visualizing and understanding how ligands interact within their target environments, often highlighting key bonds and interactions such as hydrogen bonds and hydrophobic contacts. The detailed findings of this analysis were systematically presented in Supplementary Table S2 and visually illustrated in binding site prediction through the Discovery studio (Figure 4), 2D interactions (Figure 5) and Intermolecular interactions through the LigPlot analysis (Figure 6). These figures and tables likely provide a comparative visual representation of the interaction patterns of each ligand, offering insights into their binding affinities and potential efficacy. Such analyses are crucial in drug discovery and development, aiding in the identification of promising lead compounds based on their molecular interactions. In this study of intermolecular interactions, the comparison of ligand interactions with the 1CEQ and 4ZL4 protein complexes reveals interesting insights. For the 1CEQ complex, scillarenin (ligand 100) formed 8 hydrophobic contacts and 1 hydrogen bond, whereas standard chloroquine showed higher hydrophobic interactions with 12 contacts. For the 1CEQ complex, 4-[(Z)-(6-Hydroxy-3-oxo-1-benzofuran-2(3H)-ylidene) methyl] phenyl beta-D-glucopyranoside (ligand 165) demonstrated a significant affinity by establishing 8 hydrophobic contacts and 6 hydrogen bonds, indicating a robust interaction profile. Common interaction sites for ligand 100 and chloroquine included Gly29, Met30, Ile31, Thr97, Ala236, and Pro246, while ligand 165 and chloroquine also have common interactions like Asn140, His195, and Gly196. Similarly, for the 4ZL4 complex, scillarenin formed 7 hydrophobic contacts, chloroquine complex with 4ZL4 had 11, and 4-[(Z)-(6-Hydroxy-3-oxo-1-benzofuran-2(3H)-ylidene) methyl] phenyl beta-D-glucopyranoside (ligand 165) complex with 4ZL4 exhibits 12 hydrophobic contacts and 2 hydrogen bonds. These findings highlight the variability in ligand-protein interactions depending on the specific protein structure and the chemical nature of the ligands involved.

The observed anti-malarial activity of lead compounds is often influenced by a combination of structural features such as hydrogen bond donors and acceptors, aromatic rings, and hydrophobic regions. Hydrogen bond donors and acceptors play a crucial role in forming strong interactions with the active sites of target proteins in malarial parasites, potentially inhibiting their function. Aromatic rings contribute to the ability of the molecule to engage in π–π stacking interactions, which enhances its binding affinity and specificity to the target. Furthermore, the hydrophobic regions in the compound facilitate interactions with nonpolar residues in parasite proteins, thereby increasing the overall binding strength and efficacy of the compound (Chen et al., 2018; Silva-Álvarez et al., 2015). The interplay of these structural elements allows the optimization of the pharmacodynamic and pharmacokinetic properties, ultimately driving the antimalarial activity of lead compounds. The hydrophobic regions in compounds play a crucial role in enhancing their interactions with nonpolar residues in parasite proteins, as demonstrated by the data. For instance, ligand 100 engages with hydrophobic residues, such as ALA236, PRO246, and ILE31, in the 1CEQ target protein through alkyl and Pi-alkyl interactions. Similarly, in the 4ZL4 target protein, interactions are predominantly hydrophobic, involving residues such as VAL153, ARG167, and PRO200, highlighting the significance of nonpolar interactions in stabilizing the ligand-receptor complex. These hydrophobic contacts are essential for binding affinity and specificity because they fortify the noncovalent interactions essential for molecular recognition. This underscores the importance of designing ligands with strategically positioned hydrophobic groups to optimize binding to parasite target proteins, thereby enhancing the effectiveness of therapeutic interventions.

### 2.8 MD simulations

**FIGURE 7:**
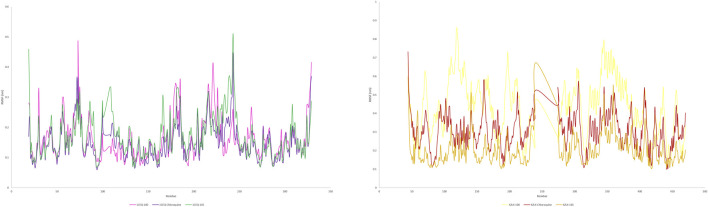
RMSD plot of protein(s) complex with standard chloroquine and lead phytoconstituents from methanolic extract of *S. oleosa* bark.

**FIGURE 8:**
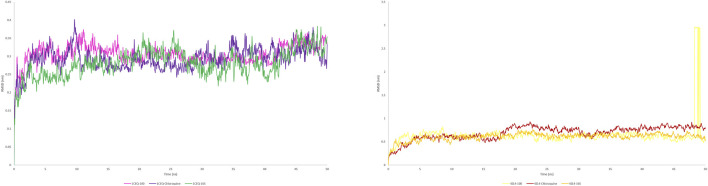
RMSF plot of protein(s) complex with standard chloroquine and lead phytoconstituents from methanolic extract of *S. oleosa* bark.

**FIGURE 9:**
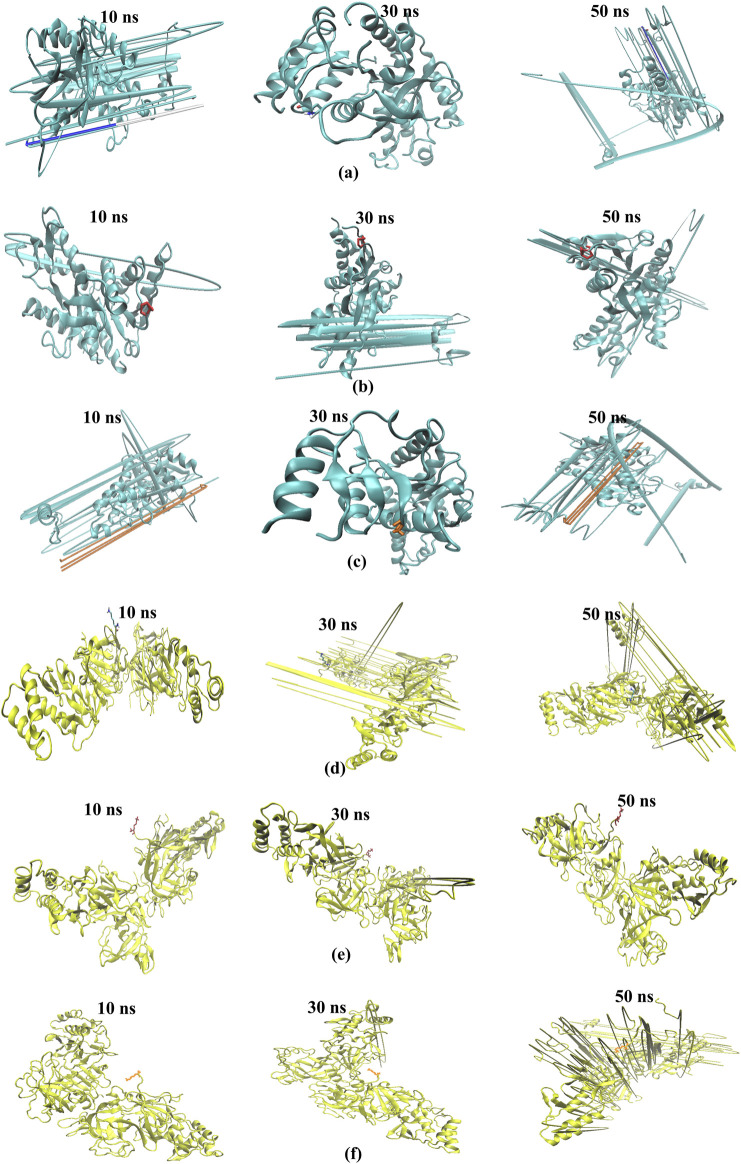
Snapshots at different intervals (time step = 10, 30, 50 ns) for the binding modes of the ligand-protein complexes. The cartoon representation shows 1CEQ–Ligand 100 **(A)**, 1CEQ–standard chloroquine **(B)**, 1CEQ–Ligand 165 **(C)**, 4ZL4 – Ligand 100 **(D)**, 4ZL4 – standard chloroquine **(E)** and 4ZL4 – Ligand 165 **(F)**.

The study aimed to evaluate the stability and interaction dynamics of protein-ligand complexes using molecular dynamics (MD) simulations, focusing on standard (chloroquine) and lead compounds scillarenin and 4-[(Z)-(6-hydroxy-3-oxo-1-benzofuran-2(3H)-ylidene) methyl] phenyl beta-D-glucopyranoside against the targets - 1CEQ and 4ZL4. The simulations, carried out over a 50-nanosecond production phase, provided insights into the structural and dynamic characteristics of these complexes. An important part of the analysis focused on evaluating the backbone root-mean-squared deviation (RMSD) and Root Mean Square Fluctuation (RMSF), which offer insights into the stability and flexibility of protein-ligand interactions. Figures 7, 8 present the RMSF plots for ligands and standard chloroquine protein complexes, revealing detailed information about the flexibility and dynamic behavior of these interactions during molecular dynamics simulations. These plots pinpoint areas of the protein with notable fluctuations, suggesting regions of flexibility or instability in the complex. Furthermore, Table 3, which illustrates the average RMSD and RMSF values, provided a quantitative overview of the stability and conformational changes experienced by the protein ligand and standard chloroquine complexes throughout the simulation. The study of ligand-protein complexes through molecular dynamics simulations provides valuable insights into their binding modes and stability over time. Snapshots were taken at intervals of 10, 30, and 50 ns to observe interactions within the different complexes. For the 1CEQ protein, three different ligands were evaluated: Ligand 100, standard chloroquine, and ligand 165. The 4ZL4 protein was analyzed using the same set of ligands. The cartoon representations of these complexes reveal distinct binding interactions and conformational changes over time. In the case of 1CEQ, ligand 100 showed a stable interaction pattern, whereas standard chloroquine and ligand 165 exhibited unique binding characteristics that could influence their efficacy. For the 4ZL4 protein, ligand 100 and standard chloroquine exhibited different binding dynamics, with ligand 165 showing potential for alternative interaction pathways. Snapshots of the ligand-protein complexes were created at specific time points (10, 30, and 50 ns) to visualize the position and binding modes of the ligands over a 50-ns MD simulation, as illustrated in Figures 9A–F. These observations will inform further research on optimizing ligand design for targeted therapeutic applications.

**TABLE 3:** The average values of RMSD and RMSF of lead hit from the bark of *S. oleosa* and standard chloroquine.

S. No	Target	Ligand or Std	RMSD (nm) Average ± SD	RMSF (nm) Average ± SD
1	1CEQ	Ligand 100	0.306 ± 0.027	0.613 ± 0.068
		Std Chloroquine	0.293 ± 0.031	0.149 ± 0.059
		Ligand 165	0.281 ± 0.038	0.171 ± 0.071
2	4ZL4	Ligand 100	0.647 ± 0.294	0.394 ± 0.146
		Std Chloroquine	0.690 ± 0.143	0.281 ± 0.098
		Ligand 165	0.606 ± 0.078	0.203 ± 0.081

Monitoring the conformational and structural changes of malarial targets complexed with lead compounds such as scillarenin and 4-[(Z)-(6-hydroxy-3-oxo-1-benzofuran-2(3H)-ylidene) using RMSD and RMSF analyses provides valuable insights into their stability and dynamics. The RMSD analysis, conducted over a 50 ns trajectory, revealed that the 1CEQ – ligand 165 complex exhibited the lowest average RMSD value of 0.281 nm, indicating high stability. Comparatively, the standard chloroquine and ligand 100 showed slightly higher RMSD values of 0.293 nm and 0.306 nm, respectively. In another set of analyses, the 4ZL4 – ligand 165 complex similarly demonstrated the lowest RMSD at 0.606 nm, suggesting better stability compared to ligand 100 and standard chloroquine, which had RMSD values of 0.647 nm and 0.690 nm respectively. These findings suggest that ligand 165 might have a more stable binding conformation with both targets compared to the other compounds tested.

The RMSF analysis provides valuable insights into the dynamic behavior of proteins during molecular simulations, particularly in understanding protein-ligand interactions. By examining RMSF values, researchers can discern flexible regions within protein structures, such as 1CEQ and 4ZL4, and compare their interactions with various ligands, including lead compounds and standard chloroquine. Regions with higher RMSF values exhibit more flexibility, whereas lower values indicate stability in secondary structures. This information is crucial for assessing the energy and stability of ligand-binding interactions, which can influence the efficacy of potential drug compounds. In the specific cases of 1CEQ, significant RMSF values were observed with different ligands: ligand 165 (0.171 nm), standard chloroquine (0.149 nm), and ligand 100 (0.613 nm). Similarly, for 4ZL4, the RMSF values with ligand 165 (0.203 nm), standard chloroquine (0.283 nm), and ligand 100 (0.394 nm) reveal variations in flexibility and interaction dynamics. These findings are pivotal for designing and optimizing therapeutic agents targeting such proteins.

In comparing the results of our MD simulations of scillarenin and 4-[(Z)-(6-hydroxy-3-oxo-1-benzofuran-2(3H)-ylidene) methyl] phenyl beta-D-glucopyranoside with those of chloroquine, it is essential to contextualize our findings within the broader landscape of existing literature. MD simulations revealed that ligand 165 exhibited a lower average RMSD, indicating greater stability than chloroquine, which aligns with findings from other studies that have reported similar stability profiles for phytochemicals targeting the same protein receptors. For instance, previous MD studies have shown that certain natural compounds maintain stable interactions through robust hydrogen bonding and hydrophobic contact, similar to those observed in our simulations. However, our results also highlight unique interaction patterns, such as the additional hydrogen bonds formed by ligand 165, which may enhance its binding affinity and therapeutic potential. This comparative analysis not only underscores the promising nature of our lead compounds but also suggests that they could offer advantages over chloroquine in terms of efficacy and safety. Future research should focus on the experimental validation of these computational findings and explore additional ligands to further elucidate the mechanisms underlying their interactions with target proteins. By situating our results within the context of existing studies, we can better understand the implications of drug design and the potential for developing new antimalarial therapies.

Hydrogen bonds offer precise and directional connections between the polar groups of the ligand and complementary polar residues of the protein, thereby enhancing binding affinity through a network of stable interactions. These bonds ensure that the ligand is maintained in the optimal orientation, which is crucial for effective binding. MD simulations revealed that ligand 165 formed multiple hydrogen bonds with key residues, demonstrating increased stability compared to chloroquine. On the other hand, hydrophobic contacts occur between the non-polar regions of the ligand and hydrophobic residues of the protein, driven by the principle of minimizing exposure to water. These contacts generate a favorable entropic effect by bearing non-polar regions within the interior of the protein. In our study, the combination of significant hydrophobic contacts and hydrogen bonds in ligand 165 resulted in a synergistic effect, further stabilizing the protein-ligand complex.

Molecular simulations have witnessed a transformative shift because these techniques are closer to practical applications in drug discovery, particulate generation, and optimization. The dynamic nature of target-ligand interactions poses significant challenges, especially in the accurate prediction of the thermodynamics and kinetics of bonding. MD-based dynamic docking is a more nuanced approach than traditional static docking because it considers the flexibility and electronic structure of such interactions. However, the complexity of these simulations implies that advancing them to a point at which they can replace static docking remains a critical hurdle. Overcoming this challenge would mark a significant leap forward, enabling more accurate and efficient drug discovery processes that can better mimic real-world biological environments. Researchers are actively refining these techniques to enhance their speed and precision, thereby promising a new era of pharmaceutical development (Riccardi et al., 2018).

Molecular dynamics simulations serve as a powerful tool for evaluating the potential of lead compounds like scillarenin and 4-[(Z)-(6-hydroxy-3-oxo-1-benzofuran-2(3H)-ylidene) methyl] phenyl beta-D-glucopyranoside, particularly in comparison with standard antimalarial drugs such as artesunate and chloroquine. These simulations provide a dynamic view of how these compounds behave in a physiological setting, offering valuable insights into their stability and flexibility. The stability of a compound in a simulated physiological environment is a crucial factor in assessing its potential as a therapeutic agent, particularly for treating diseases like malaria. When compounds such as scillarenin exhibit stable interactions with target proteins during simulations, they exhibit strong binding affinity. This stability suggests that the compound can effectively maintain its inhibitory action against the malaria parasite over time, thereby enhancing its potential efficacy as a treatment.

The flexibility of lead compounds is crucial for determining their efficacy, particularly in the context of drug design and development. When a compound can adapt its conformation to more precisely fit the binding site of a target protein, it often results in more robust and specific binding interactions. This adaptability can enhance the ability of a compound to effectively inhibit or modulate a target protein’s activity, which is essential for therapeutic applications. In the case of antimalarial agents like scillarenin, their ability to undergo conformational changes while maintaining favorable interactions with the target protein can significantly contribute to their effectiveness. This flexibility allows patients to better navigate the complex environment of the human body and adapt to the dynamic nature of biological systems, ultimately improving therapeutic outcomes.

Malaria remains a significant global health challenge, particularly in regions with tropical climates where severe cases contribute to higher mortality rates. The disease not only affects the health of individuals but also imposes substantial social and economic burdens on affected communities. Although numerous natural and synthetic anti-malarial agents have been developed, the continued evolution of drug-resistant strains of the malaria parasite has hindered effective disease control. This resistance underscores the urgent need for novel and innovative therapeutic approaches that can successfully overcome these challenges (White et al., 2014). Research into new drugs, including those targeting different stages of the parasite’s life cycle or employing unique mechanisms of action, is crucial. Multidisciplinary efforts combining genomics, drug discovery, and public health strategies are essential to develop effective solutions and ultimately reduce the impact of malaria worldwide.

Herbal medicines have long been recognized as a rich source of bioactive compounds, which have the potential to be developed into new pharmaceutical drugs. The diverse chemical structures found in plants can lead to the discovery of unique mechanisms of action that traditional synthetic drugs might not offer. Moreover, the study of herbal medicines can inspire the synthesis of new compounds that mimic the beneficial properties of these phytoconstituents, leading to innovative treatments for various health conditions. As research in ethnopharmacology and phytochemistry advances, the potential for herbal medicines to contribute to modern drug development continues to grow, offering promising avenues for both prevention and treatment of diseases (Mustafa et al., 2017).

The study on the bark of *S. oleosa* provides significant insights into its potential medicinal properties, particularly its antimalarial effects. By employing advanced analytical techniques such as GC-MS and LC-MS, researchers identified 175 phytoconstituents in the bark. Among these, phenolics, flavonoids, and tannins were quantitatively analyzed, as they are known for their health benefits, including antimicrobial and antioxidant properties. The methanolic extract of the bark demonstrated promising *in-vitro* antimalarial activity, particularly against the malaria-causing Plasmodium species. The candle jar method is a simple and effective technique for cultivating microorganisms, particularly in anti-malarial *in vitro* studies. This method involves placing Petri dishes or culture flasks containing the parasite and erythrocytes inside a sealed jar with a light candle. The burning candle consumes oxygen and raises carbon dioxide levels, creating a low-oxygen, high-carbon dioxide environment that mimics the conditions found in human blood. This environment is conducive to the growth and replication of *P. falciparum*, allowing researchers to study its life cycle and test its potential as an antimalarial drug (Hanifian et al., 2022). These findings suggest that *S. oleosa* could be a valuable source for developing new antimalarial treatments, warranting further investigation and development. The methanolic extract’s average IC50 value of 0.780 μg/mL, though higher than that of standard antimalarial drugs chloroquine (0.020 μg/mL) and quinine (0.268 μg/mL), still demonstrates promising antimalarial efficacy. While a higher IC50 value generally indicates a lower potency, the extract’s ability to inhibit malaria parasites suggests it could serve as a potential alternative or complementary treatment. The bark extract, which exhibits anti-malarial properties, is generally less effective than the standard treatments of chloroquine and quinine. This diminished efficacy could be attributed to the concentration and bioavailability of active compounds in the extract.

The presence of tannins in the bark of *S. oleosa* is a significant contributor to its antimalarial properties. These phytochemicals, known for their pharmacological activity, have been extensively studied for their ability to combat malaria parasites. Research, such as that by Bhatia et al. (2013), confirms the presence of tannins in *S. oleosa*, supporting the notion that these compounds play a crucial role in the medicinal efficacy of the plant. Furthermore, recent studies, such as Paes et al. (2024), emphasize the potential of tannins as effective agents against malaria, highlighting the importance of these compounds in developing alternative therapeutic strategies. The high tannin content in *S. oleosa* may thus offer valuable insights into natural antimalarial solutions, highlighting the potential of the plant for medical and pharmacological applications.

Further research could explore its active compounds, optimize its formulation, and assess its safety and efficacy in clinical settings, potentially leading to new therapeutic options in the fight against malaria. The study of computational analyses involving molecular docking and dynamics has revealed promising insights into the potential antimalarial properties of the bark of *S. oleosa*. By targeting malaria-related proteins such as 1CEQ and 4ZL4, the research aimed to compare the effectiveness of these compounds against established antimalarial drugs like quinine and chloroquine. The results highlighted those compounds derived from *S. oleosa* exhibited the highest docking scores, suggesting a strong potential for binding and inhibition of the target proteins. Furthermore, comprehensive ADMET profiling and evaluation of drug likeness characters demonstrated the feasibility of these compounds as viable therapeutic agents. *In silico* ADMET profiling involves the use of computer-based simulations and models to evaluate the pharmacokinetic and safety profiles of compounds before they reach the costly and time-intensive stages of *in vitro* and *in vivo* testing. By employing advanced algorithms, machine learning, and molecular modeling techniques, scientists can efficiently screen large libraries of compounds and identify those with favorable ADMET characteristics. This not only accelerates the development of new pharmaceuticals and reduces the likelihood of late-stage failures, making the drug development process more cost-effective and streamlined. In silico ADMET profiling is particularly valuable for identifying potential safety concerns, optimizing lead compounds, and guiding experimental design.

The evaluation of compounds as potential drug candidates for malaria involves more than simply analyzing numerical docking scores. Although these scores provide preliminary insights into the binding affinities of compounds with malaria receptor targets, they are insufficient as standalone indicators of drug viability. To fully assess a compound’s drug candidacy, it is crucial to consider a range of factors beyond its binding affinity. ADMET properties play pivotal roles in determining the safety and efficacy of a compound in a biological context. Additionally, understanding a compound’s pharmacokinetics and pharmacodynamics, potential off-target effects, and synthetic feasibility is essential for comprehensive evaluation. Validation through rigorous *in vitro* and *in vivo* studies is necessary to confirm initial hypotheses regarding biological activity and specificity. Benchmarking these new compounds against established antimalarial drugs, such as chloroquine and artemisinin, can provide valuable information regarding their therapeutic potential. By integrating these dimensions, researchers can identify viable drug candidates and advance their development toward effective malaria treatment.

This study highlights the promising potential of phytoconstituents derived from the bark of *S. oleosa* as therapeutic agents against malaria, with a focus on docking scores. Two compounds, 4-[(Z)-(6-Hydroxy-3-oxo-1-benzofuran-2(3H)-ylidene) methyl] phenyl beta-D-glucopyranoside and scillarenin, demonstrated the lowest binding energies (high docking scores) compared with the standard chloroquine, indicating a strong affinity for the malaria target proteins 1CEQ and 4ZL4. The dual targets of these proteins demonstrate their potential effectiveness. In addition, the favorable drug-likeness properties of the compounds and their ADMET profiles indicated promising safety and efficacy profiles, which are essential for drug development. The promising characteristics of the compounds prompted further investigation through MD simulations, which helped to evaluate their stability and behavior within a simulated biological setting, yielding more in-depth information about their therapeutic potential against malaria. The exploration of natural products with anti-malarial activity has a rich history, with compounds such as artemisinin from *Artemisia annua* and chloroquine from *Cinchona calisaya* being notable successes in the fight against malaria (Dahnum et al., 2012; Carroll et al., 2012). In particular, artemisinin has revolutionized malaria treatment because of its rapid action against Plasmodium parasites. Similarly, chloroquine has been a cornerstone treatment for decades, until resistance issues emerged. These successes underscore the potential of harnessing natural phytoconstituents to combat malaria. The bark of *Schleichera oleosa*, a lesser-known botanical source, has been investigated for its similar properties. Research on *Schleichera oleosa* has identified novel compounds that could enhance existing anti-malarial regimens or provide entirely new therapeutic options. This study would benefit from including references to similar *in vitro* antimalarial studies, as this would help position the findings within the broader context of phytoconstituents research (Deshpande et al., 2014). This research highlights the significance of their findings, demonstrates how their work contributes to existing knowledge, and identifies novel aspects of their study. The recent study on the bark of *S. oleosa* has led to the groundbreaking identification of two key phytoconstituents, scillarenin and 4-[(Z)-(6-hydroxy-3-oxo-1-benzofuran-2(3H)-ylidene) methyl] phenyl beta-D-glucopyranoside, which demonstrate promising antimalarial activity. Recent *in silico* studies have identified compounds such as 4-[(Z)-(6-hydroxy-3-oxo-1-benzofuran-2(3H)-ylidene) methyl] phenyl beta-D-glucopyranoside, which were previously unreported for such activity, exhibit promising anti-malarial properties. Although scillarenin has already been noted for its antioxidant activity (Kakouri et al., 2019), this finding expands the potential therapeutic applications of these compounds. This study highlights the power of computational methods in drug discovery and provides a foundation for further experimental studies to explore the efficacy and safety of these compounds in treating malaria. This discovery marks a significant step forward in the search for new antimalarial drugs, as these compounds have shown potential in initial testing for their ability to combat the malaria parasite. The study not only highlights the potential of these bioactive constituents but also supports the traditional medicinal use of *S. oleosa*. Further *in vivo* testing is recommended to fully assess the therapeutic potential of these compounds and to develop them into effective treatments for malaria, a disease that continues to have a devastating impact worldwide.

## 3 Conclusion

The present study highlights the promising anti-malarial potential of bioactive phytoconstituents from *S. oleosa*, revealed through advanced GC-MS and LC-MS analyses. By employing *in silico* molecular docking studies, researchers identified compounds with strong binding affinities to malarial targets 1CEQ and 4ZL4, suggesting that these phytoconstituents could serve as lead molecules in drug discovery. The virtual screening methods, including ligand-based drug design and MD simulations, demonstrated that these phytoconstituents not only possess drug-like properties but also exhibit favorable pharmacokinetic profiles, reducing potential side effects and the likelihood of failure in clinical trials. The investigation into the potential antimalarial properties of chloroquine and identified phytoconstituents from the methanolic extract of *S. oleosa* bark has yielded promising results. The analysis of drug likeness, ADMET, and molecular dynamics simulations, particularly looking at RMSD and RMSF, has demonstrated the effectiveness of these compounds as potential drug candidates. The study highlighted the phytoconstituents such as scillarenin and 4-[(Z)-(6-hydroxy-3-oxo-1-benzofuran-2(3H)-ylidene) methyl] phenyl beta-D-glucopyranoside standing out as strong inhibitors against malaria targets. These results highlight the promise of these compounds in malaria treatment, indicating that further research is necessary to confirm their therapeutic potential and determine the optimal dosage for clinical use. This research is a promising step towards developing new plant-based antimalarial therapies, which could provide more effective strategies in the fight against this global health issue. The discovery of these lead compounds represents a significant advancement in the creation of new malaria medications.

## 4 Experimental

### 4.1 Chemicals

Petroleum ether, chloroform, methanol, gallic acid, hydrochloric acid (Molychem, Mumbai); folin-ciocalteu reagent, rutin, tannic acid, quercetin, sodium nitrite, sodium hydroxide (CDH, Delhi); aluminum chloride (Qualikems, Vadodara); HEPES, 1% D-glucose, 0.23% sodium bicarbonate, 10% heat-inactivated human serum, 5% D-sorbitol, 3% hematocrit and JSB stain.

### 4.2 Collection, authentication, and preparation of plant material

The process of identifying *S. oleosa* began with the meticulous collection of plant material from its natural habitat. This was followed by the preparation of a herbarium voucher specimen, cataloged as IGNTU/DOB/2024/Sap/S0/01, to ensure accurate documentation and future reference. The taxonomical features of *S. oleosa* were thoroughly examined against descriptions found in local flora, as outlined in published sources. Photographs and voucher specimens were checked against trusted botanical databases such as the Plant List, the Flora of India websites, and the International Plant Names Index (IPNI) to guarantee accurate identification. Furthermore, the taxonomy of the species was corroborated using the Germplasm Resources Information Network and other specialized databases like IMPPAT. This comprehensive approach allowed for the confirmation of the species, genera, and family classifications of *S. oleosa*, as documented by Rao et al. (2024). The final step in this identification process involved validation by a taxonomist at Indira Gandhi National Tribal University, located in Amarkantak, Madhya Pradesh, India.

The fresh bark of the *S. oleosa* plant was collected from the nursery in Bhadi, district Mandla, Madhya Pradesh, India. Drying, grinding, and storing plant material as a powder before extraction are crucial steps to ensure efficient and high-quality extraction. Drying removes moisture content, prevents the microbial growth and degradation of active compounds. This step helps to preserve the plant material and extend its shelf life. The grinding of dried plant material into a fine powder increases the surface area, enhancing the ability of the solvent to penetrate plant cells and extract desired compounds more effectively. This process also ensures more uniform extraction, which improves the yield and consistency of the active ingredients. Proper storage of the powdered material is essential to protect it from environmental factors such as light, air, and humidity, which can further degrade the compound (Krakowska-Sieprawska et al., 2022). The recently collected bark was cut into small pieces and allowed to air dry under shade for 10 days and was pulverized into a coarse powder using an electric blender. Unwanted debris was removed and weighed on a scale balance to know the initial weight of the powder. For later use, the pulverized plant material was then stored in a dry and clean bag.

### 4.3 Extraction of *S. oleosa* bark powder

Soxhlet extraction is a widely favored technique for extracting compounds from solid materials because of its efficiency, simplicity, and ability to handle various sample types. One of the primary reasons for selecting Soxhlet extraction is its ability to repeatedly wash samples with fresh solvents, thereby enhancing the extraction process without manual intervention. This method is particularly effective for extracting non-volatile and semi-volatile compounds because it allows for the continuous recycling of solvents, which ensures thorough extraction. In addition, Soxhlet extraction is compatible with various solvents, enabling researchers to select the most appropriate solvent for specific compounds (Bhadange et al., 2024). A 100 g sample of powdered *S. oleosa* bark underwent sequential extraction using petroleum ether (60°C–80°C), chloroform, and methanol with the help of a Soxhlet apparatus, resulting in their respective extracts. The process continued until the siphoning tube of the Soxhlet apparatus showed no further coloration. The obtained extracts were then processed with a rotary evaporator to eliminate the solvent from each extract. The final weight of each extract was measured, and stored under 4°C. The percentage yield was determined based on the initial weight of the powder, as demonstrated below:

$$\%\mathrm{Yield}=\frac{Final\;weight\;of\;extract}{Initial\;weight\;of\;the\;powdered\;plant\;bark}\;x\;100$$

### 4.4 Quantitative analysis

Quantitative analysis involves measuring the total quantity of active compounds, also known as secondary metabolites, in a plant sample. Each type of secondary metabolite requires a specific reference standard for accurate quantification (Phong et al., 2022).

#### 4.4.1 Determination of total phenolic content

With some adjustments, the FC reagent method was employed to measure the total phenolic content (Georgé et al., 2005a). A 100 mL volumetric flask was filled with 10 mg of accurately measured standard gallic acid to prepare a working standard (WS) solution with a concentration of 100 μg/mL. Methanol was used as the solvent to fill the flask to the mark. Specific volumes of this WS solution were then transferred into several 10-mL volumetric flasks to achieve final gallic acid concentrations ranging from 0 to 15 μg/mL, which were used to construct a calibration curve. Each flask containing different standard concentrations was supplemented with 2 mL of FC reagent, gently mixed, and allowed to sit for 2 minutes. Following this, 2 mL of a 20% Na_2_CO_3_ (w/v) solution was added, and the volume was adjusted with distilled water. The samples were then incubated in the dark for 30 min or until a blue color developed. The absorption peaks of these solutions were measured against a blank solution at 755 nm using UV-visible spectrophotometry with the UV-Probe on a UV-1900, Shimadzu (Shimadzu, Japan). This method was similarly used on samples prepared in triplicate by taking suitable portions of the sample solutions to measure their concentration. The TPC is quantified by comparing the sample data with a standard known as gallic acid, a common benchmark in phenolic studies. The results are expressed in terms of milligrams of gallic acid equivalents (GAE) per gram of the dried sample, using a gallic acid standard curve (Ahmed et al., 2017; Assefa and Keum, 2017; Georgé et al., 2005b; Rao et al., 2024).

Total phenolic content mg GAE/g=c.VM

Where C – is the regression equation’s predicted amount of gallic acid (mg/mL); V – volume of extract used for analysis (mL); M - Weight of extract used for analysis (g).

#### 4.4.2 Determination of total flavonoid content

With only slight adjustments, the colorimetric technique was employed to assess the total flavonoid content (TFC) of the extract using aluminum chloride (AlCl_3_) (Matvieieva et al., 2019a). A 100 mL volumetric flask was used to prepare a working standard (WS) solution by adding 10 mg of precisely measured standard rutin, resulting in a concentration of 100 μg/mL. Methanol served as the solvent to fill the flask to the required volume. The described procedure outlines a method for constructing a calibration curve for rutin concentration determination using spectrophotometry. To begin, various aliquots of a working solution were precisely transferred into 10 mL volumetric flasks to achieve rutin concentrations varying from 0 to 25 μg/mL. This range enables the creation of a detailed calibration curve. Each flask was supplemented with 0.3 mL of a 5% sodium nitrite (NaNO_2_) solution, followed by a gentle shake and a 10-min incubation to allow for any necessary reactions. Subsequently, 0.3 mL of a 10% AlCl_3_ solution was added to each flask, and another incubation period was observed. To complete the process, 2 mL of a 1 M sodium hydroxide (NaOH) solution was added, and the final volume was adjusted with distilled water. The absorbance of each prepared sample was measured at a wavelength of 419 nm using a Shimadzu UV/Probe 1900 UV/visible spectrophotometer. For sample analysis, appropriate aliquots of the extracts were prepared, and the procedure was consistently applied to all sample solutions in triplicate. The Total Flavonoid Content (TFC) was determined as outlined in the Total Phenolic Content (TPC) section, with results expressed in milligrams of Rutin Equivalents (mg RE) per gram of sample extract (Faujdar et al., 2019; Matvieieva et al., 2019b; Rao et al., 2024).

#### 4.4.3 Determination of total tannin content

The plant extract’s total tannin content was assessed with tannic acid serving as the reference standard (Hagerman and Butler, 1978). A 100 mL volumetric flask was carefully filled with 10 mg of accurately measured standard tannic acid to prepare a working standard (WS) solution with a concentration of 100 μg/mL. Methanol served as the solvent to adjust the volume to the desired level. Specific aliquots of this WS solution were transferred into several 10-mL volumetric flasks, creating final tannic acid concentrations between 0 and 25 μg/mL, intended for a calibration curve. To each 10 mL flask containing these different standard concentrations, 0.5 mL of FC reagent was added. Following this, 1 mL of a 35% Na_2_CO_3_ solution was introduced into each flask and gently mixed for 5 minutes. The solution was then placed in a dark setting for 30 min. The plant extract was processed similarly to produce the sample solution. The absorbance of each standard solution was measured at 419 nm using the Shimadzu UV/Probe 1900 UV-visible spectrophotometer, with a blank reference. This procedure quantified the TTC as milligrams of Tannic Acid Equivalents (TTE) per gram of dried plant extract. (Muddathir and Mitsunaga, 2013; Rao et al., 2024).

### 4.5 GC-MS and LC-MS instrument and chromatographic conditions

Gas chromatography-mass spectrometry (GC-MS) is a powerful analytical technique for the identification and quantification of compounds in complex mixtures. One of the primary reasons for selecting GC-MS is its ability to provide both qualitative and quantitative data with high sensitivity and specificity. The combination of gas chromatography, which separates volatile and semi-volatile compounds, with mass spectrometry, which identifies them based on their mass-to-charge ratio, allows for the precise analysis of diverse samples. GC-MS is particularly advantageous in fields such as environmental analysis, forensic science, and pharmaceuticals because of its capacity to detect trace levels of substances and its robustness in handling complex matrices (Beale et al., 2018). GC-MS analysis of the samples was meticulously conducted using the GC-MS-QP2010 Plus system. This GCMS instrument was equipped with a 5-ms VF-fused silica capillary column of length 30 m, diameter 0.25 mm, and film thickness. Helium (99.99%) was used as the carrier gas at a constant flow rate of 1.5 mL/min. Initially, the oven temperature was maintained at 50°C for 1 minute, then methodically increased to 300°C over a period of 10 min at a rate of 7.5°C/min. The sample injector was precisely controlled at 280°C, while a 2.0 mL volume of the analytical solution was injected with a split ratio set to 1:3. The system utilized highly pure helium as the carrier gas to ensure optimal performance of the GC apparatus. The interface was consistently kept at 300°C, while the ion source was maintained at a slightly lower temperature of 250°C, optimizing conditions for efficient ionization of the analytes. The mass spectrometer operated in acquisition mode, which facilitated a comprehensive scan across a mass-to-charge (m/z) range of 50–1000. This wide range was crucial for capturing the complete fragmentation patterns of the target analytes, enabling detailed quantitative analysis. By utilizing a full scan, the process ensured that all relevant data points were collected, allowing for precise identification and quantification of the compounds under study (Patel et al., 2010). The detection was performed using the National Institute of Standards and Technology (NIST) mass spectral library. The NIST mass spectral library is a comprehensive tool for identifying chemical compounds using mass spectrometry. This library contains an extensive collection of mass spectra, which are unique fingerprints of compounds based on their mass-to-charge ratios. Mass spectrometry analysis of a sample yields a spectrum that can be matched against NIST spectra. Peak alignment is a crucial step in this process because it ensures that the observed peaks in a sample spectrum are correctly aligned with the reference spectra for accurate identification. Deconvolution processes were employed to separate overlapping peaks, which can occur when multiple compounds are present in a sample, making it possible to discern individual components. The identification criteria typically involve comparing the experimental spectrum to the library spectra using metrics such as spectral similarity scores to determine the best match.

Liquid Chromatography-Mass Spectrometry (LCMS) is a powerful analytical technique that is widely used because of its ability to separate, identify, and quantify complex mixtures with high precision and sensitivity. One primary reason for choosing LCMS is its versatility in handling a vast range of chemical compounds, from small to large biomolecules, making it invaluable in fields such as pharmaceuticals, environmental analysis, and proteomics. Additionally, LC-MS offers excellent spectrometry because of its dual-phase operation: liquid chromatography effectively separates compounds, whereas mass spectrometry provides detailed molecular information through mass-to-charge ratio analysis. This combination allows the accurate identification and quantification of compounds in trace amounts (Bhole et al., 2020). During liquid chromatography, compounds are separated by their interactions with a stationary phase and a mobile phase, allowing for the isolation of individual components within a mixture. The Waters Alliance HT 2795 system, paired with the Micro mass Quattro Micro, offers a robust solution for analyzing complex mixtures. LC-MS was coupled with a Micromass QTOF Micro Mass Spectrometer (Waters, Milford, MA, United States). Separation was performed on an XBridge C18 column (130 Å, 3.5 µm, 4.6 mm × 150 mm; Waters). The mobile phase was composed of 80% methanol and 20% water and was run in isocratic mode. The flow rate was set at 0.7 mL/min. The system’s sensitivity and precision arise from its ability to operate in positive ion mode with an electrospray ionization (ESI) source. This configuration allows for the effective ionization of analytes, which are then channelled through the mass spectrometer for analysis. The following changes have been made to the settings: the source temperature is maintained at 100°C to facilitate the efficient evaporation of solvents, while the temperature drop is set to 400°C to promote desolvation. The airflow settings, with a reduced airflow of 500 L/h and cone airflow of 50 L/h, are fine-tuned to stabilize the ion stream. The choice of argon as the collision gas ensures effective fragmentation of ions, critical for detailed mass analysis. MassLynx 4.1 is an advanced software tool that facilitates data processing and interpretation in mass spectrometry. The MassLynx 4.1 software provides a comprehensive suite of tools for accurate and efficient analysis of complex datasets. Intuitive data processing and visualization capabilities allow users to effectively interpret mass spectra and chromatograms. This process ensures that the peaks are accurately matched across the datasets, accounting for shifts that may occur due to experimental variations. In addition, MassLynx 4.1 offers robust deconvolution processes, which enable users to resolve complex spectra into individual components. This software is particularly useful for identifying and quantifying overlapping peaks, thereby enhancing the clarity and accuracy of analysis. In addition, the software provides comprehensive identification criteria, allowing users to set specific parameters for peak identification, such as retention time, mass accuracy, and intensity thresholds.

This software integrates seamlessly with both Liquid Chromatography (LC) and Mass Spectrometry (MS), creating a powerful hyphenated technique for comprehensive chemical analysis. The LC component of this system plays a critical role in isolating and separating chemical substances within a complex mixture. This separation is achieved by exploiting the differing affinities of the compounds to the stationary phase and the solvent system, allowing for precise analysis of individual components. MS component provides an in-depth examination of the ionized particles by measuring their mass-to-charge (m/z) ratios. This detailed analysis generates mass spectra, which are essentially fingerprints of the molecular entities present in the sample. The mass spectra not only reveal the molecular weights but also offer insights into the structural characteristics of the sample’s constituents, thus enabling researchers to identify unknown compounds, determine the purity of a sample, and understand the molecular structure of complex substances. Data collection and processing were performed using Masslynx software (Mas-sLynx 4.1, Waters). It is a hyphenated technique, such as LC and MS, where LC is a separation technique that separates compounds in a mixture based on their affinity for stationary and solvent (mobile phase) systems. MS is a technique that analyzes the mass-to-charge (m/z) ratio of ions. MS spectra show the molecular weights and structural properties of the various components of the samples.

### 4.6 *In vitro* testing of extract for antimalarial activity

The *in vitro* antimalarial test described utilized 96-well microtiter plates, a widely recognized technique for assessing the efficacy of antimalarial compounds. This method, pioneered by Rieckmann and colleagues at Microcare Laboratory and TRC, provides a standardized approach for evaluating the growth inhibition of *Plasmodium falciparum*, a parasite that causes malaria. The 3D7 strain of this parasite was cultured in a carefully prepared RPMI-1640 medium. This medium was enriched with 10% heat-inactivated human serum to provide essential growth factors, 1% D-glucose as an energy source, 0.23% sodium bicarbonate for maintaining pH balance, and 25 mM HEPES as a buffering agent. The parasites of *P. falciparum*, initially in an asynchronous state, were synchronized to produce only ring-stage infected cells after treatment with 5% D-sorbitol.

The use of Jaswant Singh Bhattacharya (JSB) staining in this assay is crucial for accurately measuring the initial ring stage parasitemia, which ranges from 0.8% to 1.5% at a hematocrit of 3% within a 200 µL volume of RPMI-1640 medium. This method ensures that parasitemia is consistently sustained by incorporating 50% O+ red blood cells into the culture. To evaluate the efficacy of different test samples, stock solutions were initially prepared at a concentration of 5 mg/mL in DMSO. These solutions were subsequently diluted with the culture medium to achieve the desired concentrations for testing. To obtain final concentrations ranging from 0.4 μg/mL to 100 μg/mL after a fivefold dilution, 20 µL of the diluted samples were introduced into duplicate wells that contained parasitized cells. The culture plates were then incubated at 37°C in a candle jar to create the ideal conditions for parasite proliferation. After incubation, thin blood smears were made and stained with JSB stain to allow for detailed microscopic analysis. Results are given as mean value of triplicate analyses of each sample and the MIC was determined as the lowest concentration that completely inhibited schizont development. Chloroquine was used as the reference drug.

### 4.7 *In silico* experiment

#### 4.7.1 Hardware specification

The study leveraged the computational power of an AMD Ryzen 5 5500U processor, complemented by a Radeon graphics processor, and operated on the Windows 11 to ensure efficient handling of tasks and data-intensive processes.

#### 4.7.2 Software specification

The receptor protein’s structure can be downloaded from the RCSB website. Receptor screening was conducted using AutoDock software (MGL Tools 1.5.7), which was obtained from the Scripps Center for Chemical Biology’s site. The structures of the compounds identified by GC-MS were created using Chem Draw 19.1, developed by Perkin Elmer in Waltham, Massachusetts. Molecular docking was carried out through Auto Dock Vina, which is available at the Scripps Vina website. To validate the molecular docking results, the Discovery Studio Visualizer from 2dsbiovia.com was used. SMILES codes were converted online via the NCI’s Cactus server. Admet SAR, available at the East China University of Science and Technology’s website, was utilized for *in silico* studies, and LigPlot analysis was performed using LigPlot + V 2.1, received academic license from the European Bioinformatics Institute in Hinxton, United Kingdom. By using the CHARMM-GUI web server, researchers can effectively solvate these complexes, preparing them for molecular dynamics (MD) simulations. CHARMM-GUI streamlines the process, allowing for the generation of input files tailored for various simulation environments. Once the protein-ligand complexes are solvated, MD simulations can be performed using software like VMD and NAMD. VMD (Visual Molecular Dynamics) provides a robust interface for visualizing and analyzing molecular systems, while NAMD (Nanoscale Molecular Dynamics) is renowned for its high-performance simulation capabilities.

### 4.8 Preparation of the receptor

The Protein Data Bank (PDB) supplied the three-dimensional crystal structures for two enzymes that combat malaria. These proteins were visualized using Discovery Studio 2021. All water molecules were stripped from the protein, and Kollman charges along with polar hydrogens were incorporated. AutoDock was utilized to develop the grid map and identify the protein’s active site. The grid centers, specified by coordinates (x, y, and z), along with their respective receptors, are as follows: 1CEQ at (25.585, 27.077, 9.299) and 4ZL4 at (3.020, 71.923, 36.935) (Sazed et al., 2021)

### 4.9 Preparation of ligand

All the identified 175 phytoconstituents were considered for the ligand preparation process, involved several steps, such as structural modifications and adjustments, lead optimization and removal, and variations in structures. ChemDraw was utilized to create the ligand’s structure, which was then converted into a 3D mol format (Suri and Naik, 2012). The ligand preparation process is a critical step in computational chemistry and drug design, involving a series of meticulous transformations to ready a molecule for simulation or analysis. This process begins with the addition of hydrogen atoms, ensuring that all potential binding sites are satisfied, followed by the removal of heteroatoms that may interfere with the model’s accuracy. Neutralization of charges is essential to maintain a balanced system, while the formation of ionization states and tautomers allows for the exploration of different chemical possibilities. Filtration is employed to refine the dataset by removing irrelevant or redundant entries, and consideration of alternative chiralities ensures that all stereochemical configurations are accounted for. Optimization of geometries and identification of low energy ring conformers is crucial for achieving the most stable molecular structure (Usha et al., 2013). Docking studies utilize scoring algorithms to implicitly screen a chemical database, enabling the prediction of the most effective binders.

### 4.10 Molecular docking studies

Molecular docking analysis is a computational method employed to anticipate the interaction between a protein receptor and potential ligands, typically small molecules or drug candidates. This method is crucial for understanding how these molecules fit together in three dimensions, providing insights into their binding affinity and potential efficacy as therapeutic agents. In the described study, Dock Vina, a popular docking software, was employed to analyze 175 phytoconstituents derived from *S. oleosa*, identified through GC-MS/LC-MS analysis. These compounds were docked against specific protein targets, namely, 1CEQ and 4ZL4, to assess their potential as inhibitors or binders. For comparison, known antimalarial drugs, Artesunate and Chloroquine, were also docked under identical conditions. The primary aim was to evaluate the binding energy and molecular interactions between the phytoconstituents and the receptors to identify promising candidates for further biological testing. Through this computational approach, researchers can efficiently screen large compound libraries and focus on the most promising candidates for subsequent experimental validation (Faujdar et al., 2019; Maurya et al., 2023).

### 4.11 In silico ADMET analysis of selected phytochemicals

The SMILES representations of the finalized ligands were generated through the Chemdraw, and were submitted to druglikeness and ADMET (Absorption, Distribution, Metabolism, Excretion, and Toxicity) prediction programs, such as Druglikeness (http://scfbio-iitd.res.in) and AdmetSAR (https://lmmd.ecust.edu.cn). These computational tools are essential in the early stages of drug discovery because they provide insights into the pharmacokinetic and pharmacodynamic properties of compounds. In the pursuit of discovering new antimalarial therapies, researchers are increasingly focusing on phytochemicals and potential efficacy. When a phytochemical demonstrates a higher docking score than standard antimalarial drugs, it becomes a candidate for further ADMET analysis. This comprehensive evaluation considers several factors critical to drug development. MR (Molar refractivity) provides insight into the molecular volume and polarizability, while MW (Molecular weight) is crucial for ensuring the compound can be efficiently absorbed by the body. HBD and HBA (Hydrogen-bond donors and acceptors) are vital for understanding the compound’s ability to form hydrogen bonds, influencing its interaction with biological targets. LogP, the partition coefficient, indicates the compound’s hydrophilicity or lipophilicity, which affects absorption and distribution. Adherence to RO5 (Lipinski’s rule of five) ensures the compound has properties consistent with oral bioavailability. PPB (Plasma Protein Binding) assesses how much of the drug will be available in the bloodstream, and WS (Water solubility) is essential for absorption and bioavailability. Together, these analyses help identify phytochemicals with promising drug-like properties for further development as potential antimalarial agents (Ganesan, 2008; Subash and Kareti, 2021). The *in silico* predictions are followed by successful *in vivo* experiments, it underscores the reliability and accuracy of the ADMETSAR model used (Abdou et al., 2024).

### 4.12 Intermolecular interactions

The methanolic extract of the bark of *S. oleosa* has been studied for its lead phytoconstituents using advanced computational tools like Discovery Studio Visualizer and LigPlot+. These tools are instrumental in understanding molecular interactions, especially in the context of protein binding sites. By utilizing Discovery Studio Visualizer, scientists can identify important contact points and interactions that play a critical role in the binding affinity and specificity of the protein-ligand complex. This visualization facilitates a deeper understanding of the molecular mechanisms involved, aiding in the design and optimization of new compounds for drug discovery or other biochemical applications. LigPlot+, on the other hand, provides 2D schematic diagrams that highlight hydrogen bonds and hydrophobic interactions, complementing the 3D visualizations from Discovery Studio to give a comprehensive view of the binding interactions (Jain et al., 2022; Saliu et al., 2021) LigPlot analysis is an essential tool in computational biology for visualizing the interactions between ligands and target macromolecules, such as proteins. By transforming intricate three-dimensional structures into clear two-dimensional diagrams, LigPlot simplifies the understanding of how ligands—small organic molecules, peptides, or biologically active compounds—interact with their target proteins. This visualization is particularly valuable to biochemists, pharmacologists, and drug designers as it reveals critical information about the binding properties and affinities of these compounds. The graphical maps produced by LigPlot highlight different types of interactions, such as hydrogen bonds, ionic interactions, and hydrophobic contacts, with the ligand centrally positioned. These maps not only elucidate the spatial arrangement of functional groups crucial for binding but also facilitate comparative analysis across various ligand-target complexes. This understanding is fundamental to optimizing the therapeutic efficacy of these compounds, advancing drug design, and enhancing biochemical research (Subash and Kareti, 2021)

### 4.13 MD simulation

NAMD is a powerful tool for conducting molecular dynamics (MD) simulations, allowing researchers to study the complex movements and interactions of biomolecules over time. By leveraging the CHARMM-GUI online platform, scientists can streamline the process of preparing simulation inputs, particularly in generating ligand topology files. This platform offers a user-friendly interface and a suite of tools designed to facilitate the integration of NAMD’s input generator. The collaboration between these resources provides a robust framework for evaluating the optimal binding energy of ligand-protein complexes (Thandivel et al., 2024; Le et al., 2016). The CHARMM-GUI server is an invaluable tool for researchers conducting MD simulations, particularly when it comes to constructing ligand and standard topologies. In this specific study, K+ and Cl-ions were incorporated to neutralize the systems, which were then solvated in water. The systems underwent a rigorous equilibration process using the standard CHARMM-GUI Membrane Builder protocol, starting with energy minimization through the steepest descent method for 1000 steps. This was followed by equilibration steps under NVT and NPT ensembles for 100 ps each, leading up to a comprehensive 50-nanosecond MD simulation. The relatively short timescale of 50 ns and idealized simulation conditions often used in computational studies can pose significant limitations on the accuracy and applicability of the results. In molecular dynamics simulations, for example, a 50 ns timescale may only capture transient phenomena or initial stages of molecular interactions, potentially missing slower, more complex processes that occur over longer periods. Idealized conditions, such as simplified molecular models or perfect environmental settings, can further limit the realism of the simulation because they might not account for variables such as temperature fluctuations, impurities, or real-world constraints. Consequently, while these simulations can provide valuable insights and theoretical frameworks, it is crucial to complement them with longer-timescale studies and experimental data to ensure comprehensive understanding and applicability to real-world scenarios (Huggins et al., 2019). The subsequent analysis of the docked ligand-target complex yielded valuable insights into the molecular interactions between the enzyme and its target. This approach not only enhances the accuracy of docking results but also provides a detailed assessment of the system’s stability, contributing to a deeper understanding of the underlying molecular mechanics (Eastman et al., 2017). To determine the best configuration, a high-throughput dynamic simulation approach is necessary for examining the ligand-target receptor binding process during differentiation.

## Data Availability

The raw data supporting the conclusions of this article will be made available by the authors, without undue reservation.
