# Supplementary material for: Metabolite profiling, antimalarial potentials of Schleichera oleosa using LC-MS and GC-MS: in vitro, molecular docking and molecular dynamics
Source: Front Mol Biosci. 2025 Feb 14;12:1543939. doi: 10.3389/fmolb.2025.1543939 (PMC11867967; doi:10.3389/fmolb.2025.1543939)
Supplement: Supplementary file 1 [file Table1.docx]

**Supplementary Table 1:** GC-MS and LCMS detected phytoconstituents in the methanolic extract of the bark of *Schleichera oleosa* and their binding energy with two malarial receptor targets.

| **Compound No.** | **Name of the compound** | **Binding energy (Kcal/mol)** | |
| --- | --- | --- | --- |
|  |  | **1CEQ** | **4ZL4** |
|  | **GCMS identified compounds** | | |
| 1 | 4-{2-[(4-amino-1,2,5-oxadiazol-3-yl) oxy] ethoxy}-1,2,5-oxadiazol-3-amine | -6.4 | -6.5 |
| 2 | 2-butanol, 3-phenyl-, (R- oder S-) | -5.2 | -5.7 |
| 3 | Benzene, 1,4-dimethyl- | -5.1 | -5.6 |
| 4 | 2-Oxazolamine, 4,5-dihydro-5- (phenoxy methyl)-N-[(phenylamino)carbonyl]- | -7.7 | **-8.4** |
| 5 | Propanenitrile, 3-(dimethylamino)- | -3.3 | -3.4 |
| 6 | Piperazine, 2-methyl- | -4 | -4.1 |
| 7 | Ethanol, 2-[(2-ethylhexyl) oxy]- | -4.2 | -4.6 |
| 8 | 2-(3,5-Dimethyl-1-pyrazolyl) succinic acid | -5.6 | -6.2 |
| 9 | 2-Butanone, 4-hydroxy-3,3-dimethyl-4-phenyl- | -5.8 | -6.2 |
| 10 | Cyclopentane, butyl- | -4.4 | -4.6 |
| 11 | 9,12,15-Octadecatrienoic acid, (2-phenyl-1,3-dioxolan-4-yl) methyl ester | -7 | -5.9 |
| 12 | 2-Butenoic acid, 2-methyl- | -4.4 | 4.7 |
| 13 | Silicic acid (h4sio4), tetraethyl ester | - | - |
| 14 | L-Mannose, 6-deoxy-, diethyl mercaptal | -4.9 | -5.5 |
| 15 | Benzene, 1,3,5-trimethyl- | -4.7 | -5.4 |
| 16 | Acetamide, 2-(2-hydroxyethoxy)- | -4.1 | -4.3 |
| 17 | Glycerine | -3.6 | 4 |
| 18 | Tridecane | -4 | -4.4 |
| 19 | Phenol, 2-methoxy- | -4.8 | -4.9 |
| 20 | Benzene, 1,2,4,5-tetramethyl- | -5 | -5.6 |
| 21 | Benzene, 1-methyl-2-(1-methylethyl)- | -4.9 | -5.4 |
| 22 | Permethylated and reduced product of degradation product from H3-glycolipid by l-l-FUC | - | - |
| 23 | 2,4-Dimethylstyrene | -4.9 | -5.7 |
| 24 | Benzene, 1-methyl-2-(2-propenyl)- | -4.8 | -5.4 |
| 25 | Decane-1,2-D2 | - | - |
| 26 | 1,3-Cyclohexanediol, diacetate, trans- | -5.1 | -6.1 |
| 27 | Cyclohexanol, 5-methyl-2-(1-methylethyl)-, (1. alpha.,2. beta.,5. alpha.)- (. +/-.)- | -4.9 | -6.1 |
| 28 | 1,3-Methylene-d-arabitol | -4.9 | -5.1 |
| 29 | Decane, 3,8-dimethyl- | -3.9 | -4.5 |
| 30 | Boroxin, triphenyl- | - | - |
| 31 | Oxazolidin-2-one, 4-hydroxy-3,4,5-trimethyl-5-phenethyl- | -6.5 | -7.4 |
| 32 | 1-Hexene, 3,5,5-trimethyl- | -4.1 | -4.6 |
| 33 | 1,4:3,6-Dianhydro-. alpha. -d-glucopyranose | -4.6 | -5.5 |
| 34 | Glutaric acid, 2,7-dimethyloct-5-yn-7-en-4-yl isohexyl ester | -5.6 | -6.1 |
| 35 | Cyclohexan-1,4,5-triol-3-one-1-carboxylic acid | -5.5 | -6 |
| 36 | 6-Octen-1-yn-3-ol, 3,7-dimethyl- | -4.8 | -4.8 |
| 37 | Decanal | -4 | -4.4 |
| 38 | Methanone, 1,3-dithian-2-ylphenyl- | -5.5 | -6 |
| 39 | 7-Oxabicyclo [4.1.0] hept-3-en-2-one, 5-hydroxy-3-(hydroxymethyl)- | -4.8 | -5.6 |
| 40 | Urea, N-[2-(2-thienyl) ethyl]- | -4.8 | -5.4 |
| 41 | 2-Butenal, 2-methyl-, dimethylhydrazone | -4.3 | -4.5 |
| 42 | 1,2,3,4-Butanetetrol, [S- (R*, R*)]- | -4.5 | -4.8 |
| 43 | Tridecane | -4 | -4.3 |
| 44 | 11-Methyldodecanol | -4.3 | -4.8 |
| 45 | 2-Isopropyl-5-methyl-1-heptanol | -4.5 | -5.4 |
| 46 | l-Gala-l-ido-octose | -5.2 | -5.4 |
| 47 | 2-Pentene, 2,4,4-trimethyl- | -4 | -4.4 |
| 48 | 2-(5-Pyridin-4-yl- [1,3,4] oxadiazol-2-ylsulfanyl)-propionic acid (1-methyl-3-oxo-butylidene)-H | -6.9 | -7.5 |
| 49 | 4-Hydroxy-4-methylhex-5-enoic acid, tert. -butyl ester | -4.6 | -5.4 |
| 50 | Succinic acid, 3,7-dimethyloct-6-en-1-yl isobutyl ester | -5.5 | -5.7 |
| 51 | Adenosine, 1,2-dihydro-2-oxo- | -5.5 | -5.6 |
| 52 | Tetradecane | -4.1 | 4.6 |
| 53 | 5-Azecanol | -4.8 | -6.2 |
| 54 | 1-Chlorohexadecane | -4.1 | -4.7 |
| 55 | Acetoxy acetic acid, 3-pentadecyl ester | -5.3 | -5.4 |
| 56 | 2H-bisoxireno[2,3:8,8a] azuleno[4,5-b] furan-7(3aH)-one, octahydro-3a,8c-dimethyl-6-methylen | - | - |
| 57 | Cycloheptene, 1-(1,1-dimethylethoxy)- | -5.1 | -5.5 |
| 58 | Paromomycin | -6.5 | **-8.6** |
| 59 | Norcaranic acid, methylester, SYN | -4.6 | -5.4 |
| 60 | Guanosine | -6.7 | -7.6 |
| 61 | Disulfide, 1-(1-propenyldithio) propyl, (E)- | -4.1 | -4.2 |
| 62 | Dodecane, 2,6,10-trimethyl- | -4.5 | -5.1 |
| 63 | . beta. -D-Glucopyranose, 1,6-anhydro- | -4.4 | -5.7 |
| 64 | (2E)-8-Oxo-2-nonenal # | -4.5 | -4.7 |
| 65 | 7-Octen-3-ol, 2,3,6-trimethyl- | -4.2 | -5.3 |
| 66 | 2- Chloropropionic acid, octadecyl ester | -5.2 | -5.6 |
| 67 | 1,3,2-Dithiaborolane, 2-iodo- | - | - |
| 68 | 2-Aminoethanethiol hydrogen sulfate (ester) | -4.2 | -3.9 |
| 69 | 2-Azaspiro [4.4] non-2-ylmethanamine | -4.6 | -5.8 |
| 70 | Hydroxy toluic Acid | -5.4 | -6.2 |
| 71 | Cyclohexanone, 2-(2-oxopropyl)- | -5 | -5.6 |
| 72 | 2-Methyltetracosane | -5.1 | -5.6 |
| 73 | 1,2-benzenedicarboxylic acid, diethyl ester | -5.3 | -6.1 |
| 74 | trans-4, cis-6-Dimethyl-7-oxo-8-oxabicyclo (2.2.2) octane-2-carboxylic acid | -5.8 | -6.6 |
| 75 | Card-20(22)-enolide, 3- [(2,6-dideoxy-4-O-. beta. -D-glucopyranosyl-3-O-methyl-. beta. -D-ribo-hexopyranosyl) oxy]-5,14-dihydr | - | - |
| 76 | Phenol, 3,4,5-trimethoxy- | -4.9 | -5.7 |
| 77 | 1,3,4,5-Tetrahydroxy-cyclohexanecarboxylic acid | -5.5 | -6.2 |
| 78 | 1,5-Heptadiene, 1,5-bis (trimethylsilyl-3-methylene-, 1E,5Z- | - | - |
| 79 | Mome inositol | -5.1 | -5.7 |
| 80 | 2-Furanoctanoic acid, 5-hexyltetrahydro-, methyl ester | -5.6 | -5.9 |
| 81 | Sulphurous acid, cyclohexyl methyl isobutyl ester | -5.6 | -5.6 |
| 82 | Hexadecane, 2,6,10,14-tetramethyl- | -5.2 | -6.2 |
| 83 | Dodecane, 1,1'-oxybis- | -4.5 | -4.9 |
| 84 | Carbamic acid, (. alpha. -methylbenzyl)-, 1-ethyl-1-methylbutyl ester | -5.7 | -6.7 |
| 85 | Ethyl non-2-enoate | -4.9 | -5.1 |
| 86 | 2-Hexyldodecyl propionate | -4.6 | -4.9 |
| 87 | 2-Isopropyl-5-methyl-1-heptanol | -4.3 | -5.3 |
| 88 | Octane, 1,1'-oxybis- | -4.6 | -4.5 |
| 89 | Octanoic acid, tridecyl ester | -4.8 | -4.7 |
| 90 | 1-Decanol, 2-hexyl- | -4.8 | -4.8 |
| 91 | Methacrylic acid, tetradecyl ester | -5 | -5.3 |
| 92 | Acetic acid, thiocyanato-, 1,7,7-trimethylbicyclo [2.2.1] hept-2-yl ester, exo- | -5.5 | -5.5 |
| 93 | Heptadecane, 2,6,10,15-tetramethyl- | -5.3 | -5.5 |
| 94 | Nonahexacontanoic acid | -4.5 | -5.4 |
| 95 | Trimethylsilyl [(trimethylsilyl)sulfanyl] acetate # | - | - |
| 96 | 1H-purine, 8-(methylthio)- | -4.9 | -5.2 |
| 97 | 2,5-octadecadiynoic acid, methyl ester | -4.6 | -5.7 |
| 98 | Neophytadiene | -5.5 | -4.9 |
| 99 | 3,6-Undecandione | -4.6 | -5 |
| 100 | Scillarenin | **-8.6** | **-8.3** |
| 101 | 7-Octadecyne, 2-methyl- | -4 | -5 |
| 102 | Di(Z)-non-3-enyl phthalate | -5.3 | -6.5 |
| 103 | 1-Decyl-4(1H)-pyridinimine # | -5.5 | -5.8 |
| 104 | Eicosane | -4.7 | -4.3 |
| 105 | Hexadecanoic acid, methyl ester | -4.7 | -4.8 |
| 106 | 1-[2,2,3,3-Tetramethyl-1-(3-methyl-1-pentynyl) cyclopropyl]-1-butano | -5.5 | -5.7 |
| 107 | Silicone polymer | - | - |
| 108 | Phthalic acid, butyl undecyl ester | -5.4 | -5.8 |
| 109 | 1-[2,2,3,3-Tetramethyl-1-(3-methyl-1-pentynyl) cyclopropyl]-1-butano | -5.2 | -6.1 |
| 110 | Hexadecane, 2,6,10,14-tetramethyl- | -5.4 | -5.3 |
| 111 | Cyclopentanol, 3,3,4-trimethyl-4-p-tolyl-, (R, R)- (+)- | -6.2 | -6.9 |
| 112 | Methyl 6-O-[1-methylpropyl]-. beta. -d-galactopyranoside | -5.1 | -6.2 |
| 113 | 1-Naphthalenemethanol, decahydro-2-hydroxy-2,5,5,8a-tetramethyl-, [1 | -6.2 | -6.6 |
| 114 | 9-Octadecanone | -4.2 | -5.2 |
| 115 | 2,4,4,6,6,8,8-Heptamethyl-1-nonene | -5.3 | -5.5 |
| 116 | Azacyclotridecan-2-one | -5.5 | -6.1 |
| 117 | 2-Aminopent-4-enoic acid, N-vinyloxycarbonyl-, decyl ester | -5.3 | -5.5 |
| 118 | 9,12-Octadecadienoic acid, methyl ester, (E, E)- | -5.4 | -5.3 |
| 119 | 8-Octadecenoic acid, methyl ester | -4.9 | -5.5 |
| 120 | Hexasiloxane, tetradecamethyl- | - | - |
| 121 | Methyl stearate | -5.1 | -4.5 |
| 122 | Fumaric acid, decyl 2-methylallyl ester | -5.3 | -5.5 |
| 123 | 1-Hexadecanesulfonic acid, 3,5-dichloro-2,6-dimethyl-4-pyridyl ester | -5.4 | -5.2 |
| 124 | Nonyl octacosyl ether | -4.9 | -4.3 |
| 125 | 2-Methylhexacosane | -5 | -5.7 |
| 126 | 1-Bromotriacontane | -5 | -4.1 |
| 127 | 4-Bromobutanoic acid, heptadecylic ester | -5.4 | -4.9 |
| 128 | 14-. Beta. -H-Pregna | - | - |
| 129 | Bromotrimethylsilane | - | - |
| 130 | 1-Triethylgermanium-3-methylbut-3-en-1-yne | - | - |
| 131 | L-Mannitol, 1-deoxy-, cyclic 3,4:5,6-bis(ethylboronate) 2-acetate | - | - |
| 132 | Silane, chlorodiethyl(2-ethylhexyloxy)- | - | - |
| 133 | 5,5-Diethyltridecane | -4.8 | -5.2 |
| 134 | Tetrapentacontane, 1,54-dibromo- | -8.1 | **-8.2** |
| 135 | 1-Bromodocosane | -4.8 | -5.3 |
| 136 | 1-Decanol, 2-hexyl- | -4.5 | -4.9 |
| 137 | 14-. Beta. -H-Pregna | - | - |
| 138 | Pentatriacontane | -4.6 | -4 |
| 139 | Silikonfett se30 (Grevels) | - | - |
| 140 | Tetrapentacontane, 1,54-dibromo- | -4.6 | -4.6 |
| 141 | Eicosane | -4.9 | -4.7 |
| 142 | Octatriacontyl pentafluoro propionate | -4.9 | -6 |
| 143 | Docosane | -4.6 | -5.4 |
| 144 | 17-Pentatriacontene | -4.9 | -4.3 |
| 145 | 1,19-Eicosadiene | -4.5 | -5.3 |
| 146 | Silicone oil | -5.8 | -6.6 |
| 147 | Triacontane, 1-bromo- | - | - |
| 148 | 1-Hydroxymethyl-7,7-dimethyl-bicyclo [2.2.1] heptane-2,3-dione | -5.5 | -6 |
| 149 | Heneicosane | -4.6 | -5 |
| 150 | 1,54-Dibromotetrapentacontane | -4.3 | -5.1 |
| 151 | 1-Bromotriacontane | -4.9 | -4 |
|  | **LCMS identified compounds** | | |
| 152 | dUDP | **-8.1** | -7.4 |
| 153 | (1Z,2E)-1-({[(2R,3S,4S,5R,6S)-6-{[5,7-Dihydroxy-2-(4-oxo-2,5-cyclohexadien-1-ylidene)-2H-chromen-3-yl] oxy}-3,4,5-trihydroxytetrahydro-2H-pyran-2-yl] methyl} oxonio)-3-(4-hydroxyphenyl)-2-propen-1-olate | **-8.6** | **-9.9** |
| 154 | 1-{[5-Hydroperoxy-3,4-dihydroxy-6-(hydroxymethyl) tetrahydro-2H-pyran-2-yl] oxy}-3-[(9E,12E)-9,12-octadecadienoyloxy]-2-propanyl (9E,12E,15E)-9,12,15-octadecatrienoate | -6.1 | -6.4 |
| 155 | 3-{[5-Hydroperoxy-3,4-dihydroxy-6-(hydroxymethyl) tetrahydro-2H-pyran-2-yl] oxy}-2-[(9E,12E)-9,12-octadecadienoyloxy] propyl (9E,12E,15E)-9,12,15-octadecatrienoate | -7.4 | -7.7 |
| 156 | (-)-alpha-narcotine | -7.5 | -7.2 |
| 157 | Glutathione | -8 | **-9** |
| 158 | Daidzein 7-O-glucoside | -7.1 | -7.3 |
| 159 | Aloesin | -6.7 | **-8.2** |
| 160 | 4a,5-dihydroriboflavin | - | - |
| 161 | (1S)-1,5-Anhydro-1-(5,7-dihydroxy-4-oxo-2-phenyl-4H-chromen-6-yl)-D-glucitol | - | - |
| 162 | (1xi)-1,5-Anhydro-1-(5,7-dihydroxy-4-oxo-2-phenyl-4H-chromen-8-yl)-D-glucitol | -8 | -7.5 |
| 163 | (1S)-1,5-Anhydro-1-(5,7-dihydroxy-4-oxo-2-phenyl-4H-chromen-8-yl)-D-glucitol | **-8.1** | **-8.3** |
| 164 | 5-(beta-D-Glucopyranosyloxy)-2-(4-hydroxyphenyl)-7-chromeniumolate | **-8.2** | **-9.2** |
| 165 | 4-[(Z)-(6-Hydroxy-3-oxo-1-benzofuran-2(3H)-ylidene) methyl] phenyl beta-D-glucopyranoside | **-8.1** | **-9** |
| 166 | Daidzin | - | - |
| 167 | Magnesium 3-[8-(1-hydroxy-3-methoxy-3-oxopropyl)-2,7,13,17-tetramethyl-3,18-divinyl-21H-porphin-24-id-12-yl] propanoate | -7.7 | **-8.2** |
| 168 | Isorhamnetin 3-glucoside | **-8.8** | **-9** |
| 169 | (+)-epicatechin-3-O-gallate | **-8.8** | **-9** |
| 170 | (-)-Epicatechin-3-O-gallate | **-8.7** | **-8.2** |
| 171 | (3beta,17xi)-Stigmast-5-en-3-yl D-glucopyranoside | **-8.6** | **-8.7** |
| 172 | (3beta)-Stigmast-5-en-3-yl alpha-D-glucopyranoside | -6.6 | -6.6 |
| 173 | All-trans-Nonaprenyl diphosphate | -7.6 | -8 |
| 174 | 10-Deacetylbaccatin-III | -7.3 | -7.9 |
| 175 | 1-S-[(1E)-9-(Methylsulfinyl)-N-(sulfonatooxy) nonanimidoyl]-1-thiohexopyranose | -6.3 | -7.3 |
| Std1 | Standard Chloroquine | **-8** | **-8.1** |
| Std2 | Standard Artemisinin | -6.1 | -6.5 |

**Supplementary Table 2:** Intermolecular interactions of selected malarial targets with lead phytoconstituents from the bark of *S. oleosa* and standard chloroquine.

| **Target** | **Discovery studio visualizer** | | | **LigPlot analysis** | |
| --- | --- | --- | --- | --- | --- |
|  | **Ligand**  **or Std** | **Amino acids** | **Interactions** | **Amino acids** | **Interactions** |
| 1CEQ | Ligand 100 | ALA236  ASN140  PRO246  ILE31  ILE31  GLY29  GLY29 | Pi Alkyl  Conventional Hydrogen bond  Alkyl  Alkyl  Alkyl  Conventional Hydrogen bond  Carbon Hydrogen bond | ALA236  ASN140  PRO246  ILE31  MET30  GLY29  HIS195  THR97  THR101 | Hydrophobic  Hydrophobic  Hydrophobic  Hydrophobic  Hydrophobic  Hydrogen bond  Hydrophobic  Hydrophobic  Hydrophobic |
|  | Std  Chloroquine | ALA194  HIS195  PRO141  PRO141  PRO141  PRO141  LEU113  LEU112  THR101  HIS195 | Alkyl  Carbon Hydrogen bond  Alkyl  Alkyl  Alkyl  Pi Alkyl  Alkyl  Alkyl  Carbon Hydrogen bond  Carbon Hydrogen bond | ALA194  HIS195  PRO141  LEU113  LEU112  ASN140  ARG109  THR101  GLU321  GLY196  LYS198  ASP143  MET325  THR322 | Hydrophobic bond  Hydrophobic bond  Hydrophobic bond  Hydrophobic  Hydrophobic  Hydrophobic  Hydrophobic  Hydrophobic  Hydrophobic  Hydrophobic  Hydrophobic  Hydrophobic  Hydrophobic  Hydrophobic |
|  | Ligand 165 | ILE31  ILE31  PRO246  HIS195  THR232  ASN197  VAL233  ASN197  ASN197  ASN197  ALA236 | Pi sigma  Pi Alkyl  Pi Alkyl  Carbon Hydrogen bond  Conventional Hydrogen bond  Conventional Hydrogen bond  Unfavourable Acceptor  Conventional Hydrogen bond  Conventional Hydrogen bond  Conventional Hydrogen bond  Conventional Hydrogen bond | ILE31  PRO246  HIS195  THR232  ASN197  VAL233  ALA236  THR235  ASN234  ASN140  GLY29  MET30  MET199  GLY196 | Hydrophobic Hydrophobic  Hydrophobic Hydrogen bond  Hydrogen bond  Hydrogen bond  Hydrogen bond  Hydrogen bond  Hydrophobic Hydrophobic  Hydrophobic Hydrogen bond  Hydrophobic  Hydrophobic |
| 4ZL4 | Ligand 100 | PRO200  THR111  VAL153  VAL153  ARG167  ARG167  VAL153  THR165 | Pi Alkyl  Pi Sigma  Alkyl  Alkyl  Alkyl  Alkyl  Alkyl  Conventional Hydrogen Bond | PRO200  THR111  VAL153  ARG167  SER194  PHE166  THR165 | Hydrophobic  Hydrophobic  Hydrophobic  Hydrophobic  Hydrophobic  Hydrophobic  Hydrophobic |
|  | Std  Chloroquine | SER151  GLN455  GLN212  PRO200  LYS214  ARG167  LEU169  PRO211  LEU205  PRO200 | Carbon Hydrogen Bond  Carbon Hydrogen Bond  Carbon Hydrogen Bond  Alkyl  Alkyl  Alkyl  Alkyl  Alkyl  Pi-Alkyl  Pi-Alkyl | LEU205  GLN455  LYS214  GLN212  PRO211  ARG167  PRO200  LEU213  PHE124  GLN215  GLY198 | Hydrophobic  Hydrophobic  Hydrophobic  Hydrophobic  Hydrophobic  Hydrophobic  Hydrophobic  Hydrophobic  Hydrophobic  Hydrophobic  Hydrophobic |
|  | Ligand 165 | GLN215  SER151  SER151  ARG167  ARG167  LYS456  ASP208  GLN215 | Conventional Hydrogen Bond  Carbon Hydrogen Bond  Carbon Hydrogen Bond  Carbon Hydrogen Bond  Carbon Hydrogen Bond  Pi-Cation  Pi-Anion  Pi-Donor Hydrogen Bond | SER151  ARG167  GLN215  ASP208  LYS456  LEU205  LYS168  PRO200  ASP208  PRO211  LEU169  LYS214  GLN455  LEU213 | Hydrophobic  Hydrophobic  Hydrogen bond  Hydrophobic  Hydrophobic  Hydrophobic  Hydrophobic  Hydrophobic  Hydrophobic  Hydrophobic  Hydrogen bond  Hydrophobic  Hydrophobic  Hydrophobic |
